# Maize multi-omics reveal leaf water status controlling of differential transcriptomes, proteomes and hormones as mechanisms of age-dependent osmotic stress response in leaves

**DOI:** 10.1007/s44154-024-00159-9

**Published:** 2024-03-18

**Authors:** Liangjie Niu, Wenkang Wang, Yingxue Li, Xiaolin Wu, Wei Wang

**Affiliations:** National Key Laboratory of Wheat and Maize Crop Science, College of Life Sciences, Henan Agricultural University, Zhengzhou, 450046 China

**Keywords:** Age-related changes, Old and young leaves, Osmotic stress, Phytohormone, Proteome, Transcriptome, *Zea mays*

## Abstract

Drought-induced osmotic stress severely affects the growth and yield of maize. However, the mechanisms underlying the different responses of young and old maize leaves to osmotic stress remain unclear. To gain a systematic understanding of age-related stress responses, we compared osmotic-stress-induced changes in maize leaves of different ages using multi-omics approaches. After short-term osmotic stress, old leaves suffered more severe water deficits than young leaves. The adjustments of transcriptomes, proteomes, and hormones in response to osmotic stress were more dynamic in old leaves. Metabolic activities, stress signaling pathways, and hormones (especially abscisic acid) responded to osmotic stress in an age-dependent manner. We identified multiple functional clusters of genes and proteins with potential roles in stress adaptation. Old leaves significantly accumulated stress proteins such as dehydrin, aquaporin, and chaperones to cope with osmotic stress, accompanied by senescence-like cellular events, whereas young leaves exhibited an effective water conservation strategy mainly by hydrolyzing transitory starch and increasing proline production. The stress responses of individual leaves are primarily determined by their intracellular water status, resulting in differential transcriptomes, proteomes, and hormones. This study extends our understanding of the mechanisms underlying plant responses to osmotic stress.

**Supplementary Information:**

The online version contains supplementary material available at 10.1007/s44154-024-00159-9.

## Introduction

The frequency and duration of drought have risen by 29% globally since 2000 (UN report 2022), significantly affecting crop growth and causing considerable yield loss (Gupta et al. 2020). Plants have developed complex mechanisms to respond to drought in an age- and tissue-dependent manner (Skirycz et al. 2010; Rankenberg et al. 2021; Zhang et al. 2022).

As the principal photosynthetic organs, leaves are the most susceptible to abiotic stress, especially drought. Under drought stress, plants generally exhibit tissue-specific responses by altering physiology, modifying root growth, and closing stomata to reduce leaf water loss (Gupta et al. 2020). Drought signals promote reactive oxygen species (ROS) scavenging, osmotic regulation, and cell wall remodeling. Moreover, abscisic acid (ABA), brassinosteroid, and ethylene pathways play critical roles in stress sensing (Zhang et al. 2020), by activating a variety of stress-responsive genes that trigger stomatal closure and improve water balance (Liu et al. 2023). However, the molecular regulatory mechanisms underlying drought responses in leaves are largely unclear, particularly with respect to leaf age.

Leaf stress responses are mainly determined by the developmental age (Rankenberg et al. 2021). Young leaves are significantly less affected by abiotic stress than old leaves (Bielczynski et al. 2017). Age-dependent tolerance has been observed in various plants and abiotic stresses, such as drought (Sperdouli et al. 2021), salinity (Guo et al. 2018), heat (Xiang and Rathinasabapathi 2022), irradiation (Bielczynski et al. 2017), heavy metals (Wang et al. 2018), herbicides (Pazmiño et al. 2011), and nitrogen deficiency (Safavi-Rizi et al. 2018). Moreover, senescence-induced age-related changes accelerate the loss of stress tolerance in Arabidopsis rosette leaves (Kanojia et al. 2020).

Age-related stress responses reflect a plant's adaptation strategy. Plant adaptations to abiotic stress are related to stress signaling, hormones, stress proteins, and ROS homeostasis (Zhu 2016; Gupta et al. 2020; Zhang et al. 2022). Young and old leaves reprogram transcription and translation differently under abiotic stress, leading to significant differences in physiological performance, differentially expressed genes (DEGs), differentially abundant proteins (DAPs), metabolites and hormones, and stress tolerance (Bielczynski et al. 2017; Kanojia et al. 2020; Rankenberg et al. 2021). Under heat stress, DEGs related to thermotolerance are upregulated more highly in young than in old leaves (Xiang and Rathinasabapathi 2022). The higher saline tolerance in young leaves of *Phragmites communis* is due to the higher contents of K^+^, H_2_PO_4_^−^, sugars and amino acids, and the lower contents of Na^+^, Ca^2+^, Cl^−^ and SO_4_^2−^, compared with old leaves (Guo et al. 2018). Under high light conditions, Arabidopsis young leaves are less prone to photoinhibition than old leaves, mainly because of the higher plasticity of young leaves in their anatomy and photosynthetic apparatus for adaptation to changing light conditions (Bielczynski et al. 2017).

Age-related abiotic stress tolerance of leaves often differs according to the stress type and level. For example, chilling-induced damage was more severe in young leaves of *Cucumis sativus* (Zhang et al. 2014), and similar results were observed in *Aristotelia chilensis* under prolonged drought (González-Villagra et al. 2018). Under combined biotic and abiotic stresses, Arabidopsis young leaves exhibit lower abiotic stress responses but higher biotic stress responses than older leaves, which are mediated by both ABA and salicylic acid (SA) (Berens et al. 2019). To date, age-related abiotic stress responses have mainly been investigated in the model plant Arabidopsis (Rankenberg et al. 2021). The mechanisms responsible for this phenomenon are less understood in crop plants, such as maize.

Maize is an important cereal crop and model monocot in plant biology. Drought, temperature, and salinity are the major abiotic stresses that limit global maize production (Gong et al. 2014). Although maize leaf stress responses have frequently been studied using individual or mixed leaf samples (e.g., Zhang et al. 2020; Urrutia et al. 2021; Liu et al. 2023), little is known about the mechanisms underlying differential stress tolerance across leaves in the same plant. Given the significant differences between maize (a C_4_ plant) and Arabidopsis (a C_3_ plant) in terms of genome, physiology, structure, metabolism, and stress response (Zhang et al. 2022), it is necessary to unravel the age-related stress response in maize using a multi-omics approach, which is key for crop production, and engineering stress-tolerant maize.

Here, we compared short-term osmotic stress-induced changes in the transcriptome, proteome, and hormone profiles of maize leaves at three different ages. We identified a large number of DEGs and DAPs, mostly in the old leaves, which were associated with an age-dependent stress response. Young leaves had less water loss and fewer changes in transcriptomes, proteomes, and hormones than old leaves, which probably explains the age-related osmotic stress responses in maize leaves. Our study extends our understanding of the mechanisms underlying stress responses in plants.

## Results

### Different leaves differ in sensitivity to water deficit

**Fig. 1:**
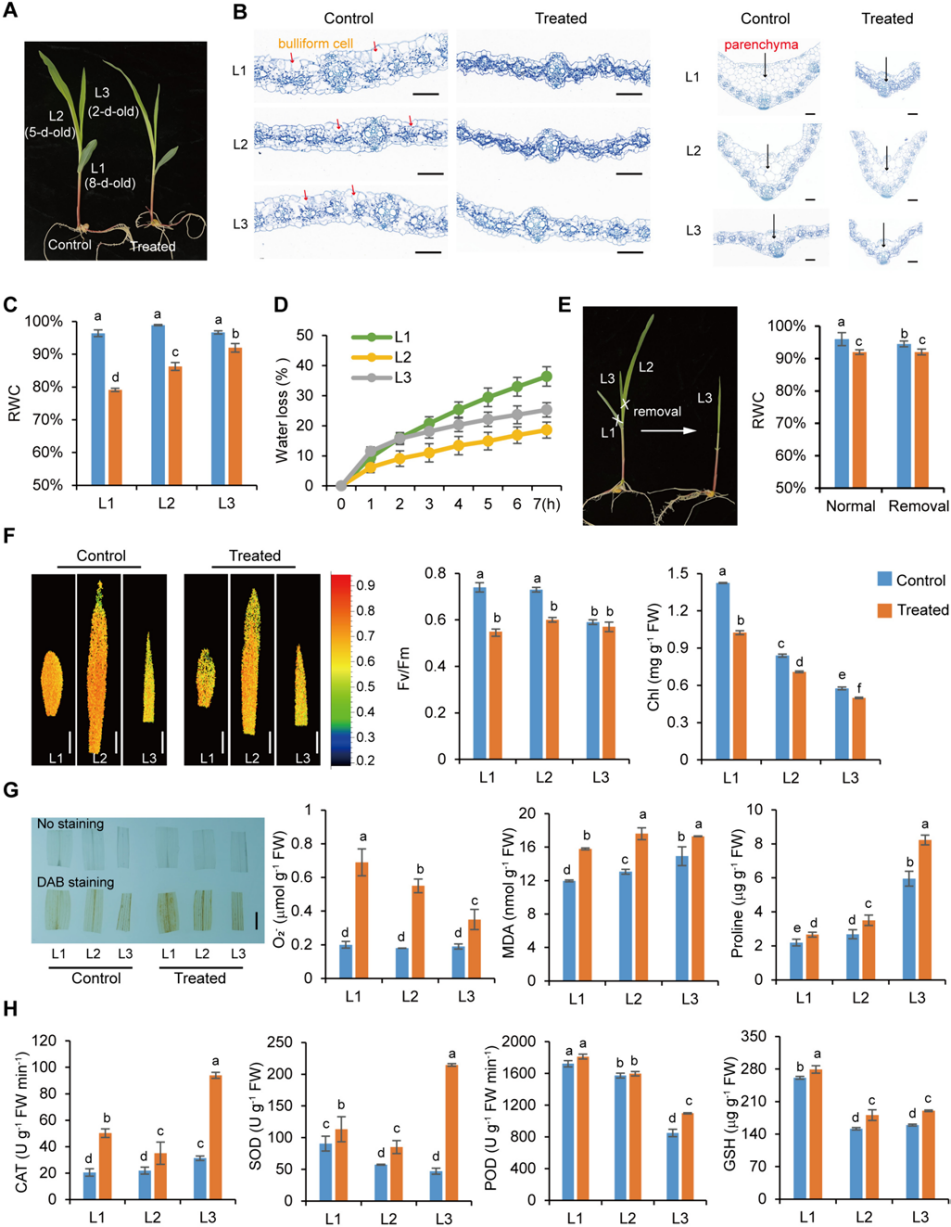
Differential physiological and biochemical changes in maize leaves of different ages under osmotic stress. Ten-d-old seedlings were subjected to 0.3 M mannitol stress for 4 h. **A** Control and treated 10-d-old seedlings with 3 visible leaves (2 ligulated). L1: the fully expanded leaf; L2, the newly-developed leaf; L3, the developing leaf. **B** Cross section of different leaves under the control and stress conditions. *Left*: cross section of leaves. *Right:* cross section of main veins. The sections were stained with safranin-fast green. Scale bars, 50 μm. **C** Relative water content (RWC) of different leaves under the control and stress conditions. **D** Water loss rate of different leaves *in vitro*. **E** RWC of L3 with or without mature leaves. **F** Measurement of Chl fluorescence, Fv/Fm, and Chl content. Scale bars, 2 cm. **G** Detection of reactive oxygen species (ROS), superoxide anions (O_2_^-^), malondialdehyde (MDA) and proline. Scale bars, 1 cm. **H** Measurement of antioxidant enzyme systems including catalase (CAT), peroxidase (POD), superoxide dismutase (SOD), and reduced glutathione (GSH). Data were obtained from at least three biological replicates. Different letters above the columns indicate statistical significance in ANOVA (*P <* 0.05) using GraphPad Prism 8.0 software

The 10-d-old seedlings of maize C7-2t had three visible leaves. L1 and L2 were mature leaves representing the source organs, whereas L3 was a rapidly growing sink organ (Fig. 1A). Osmotic stress caused initial wilting in L1, with a significant alteration in cell structure. Due to water deficit, leaf thickness decreased by 20–40% (L1 > L2 > L3), with a greatly reduced size of epidermal and mesophyll cells (Fig. 1B), while relative water content (RWC) decreased by 17% (L1), 13% (L2), and 5% (L3) (Fig. 1C). This revealed that water deficit was more severe in mature leaves than in L3 leaves under short-term osmotic stress. Leaves from maize plants of different ages were cut and used to measure water loss rates by calculating weight changes due to evaporation. The results revealed that the water loss rate progressively increased *in vitro* in the order L1 > L3 > L2 (Fig. 1D), indicating that the dehydration resistance of detached L3 declined was comparable to the *in vivo* condition. The removal of mature leaves did not affect RWC in L3 under osmotic stress (Fig. 1E). Thus, L3 had a more effective water conservation strategy than the mature leaves under osmotic stress.

Stomatal closure occurred rapidly upon osmotic stress without visible differences in stomatal parameters across the leaves (Supplementary Fig. 1). Photosynthesis was severely inhibited in mature leaves compared to L3, as indicated by decreased Chl fluorescence, Fv/Fm, and Chl content (Fig. 1F), whereas L3 maintained a similar Fv/Fm before and after stress. Stress resistance of the photosynthetic apparatus decreased sharply with increasing leaf age. L3 accumulated proline at higher levels than mature leaves under both control and stress conditions (Fig. 1G). It is well known that water deficit-induced stomatal closure leads to light capture exceeding the CO_2_ absorption rate, causing increased ROS production. L1 had higher ROS (especially O_2_^-^) levels than the younger leaves (Fig. 1G). Malondialdehyde (MDA) content increased in stressed leaves (Fig. 1G). Conversely, ROS-scavenging enzymes were elevated, especially those of catalase (CAT) and superoxide dismutase (SOD), in L3 (Fig. 1H). The content of glutathione (GSH), an important antioxidant component in plants, was increased in all stressed leaves (Fig. 1H).

Taken together, these results indicate that mature L1 and L2 were more adversely affected by osmotic stress, whereas L3 were less affected because of their more effective water conservation strategy.

## Hormone level changes in different leaves under osmotic stress

**Fig. 2:**
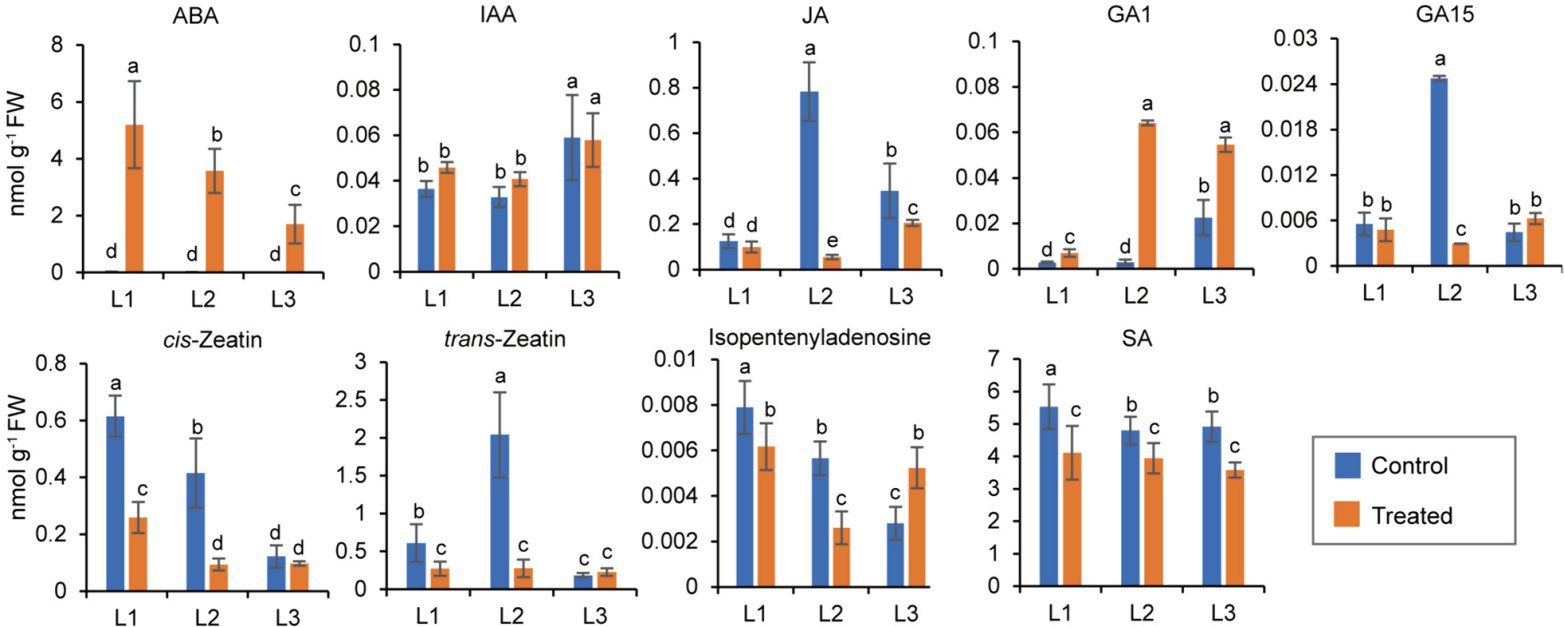
Comparison of hormone contents in different maize leaves between the control and stress conditions. Ten-d-old seedlings were subjected to osmotic stress for 4 h, and each leaf (triplicate) was used for hormone assays by LC-MS/MS. Data were obtained from at least three biological replicates. Different letters above the columns indicate statistical significance in ANOVA (*P <* 0.05) using GraphPad Prism 8.0 software

We compared the stress-induced quantitative changes in the major hormones in the leaves of three ages (Fig. 2). The ABA content was extremely low in control leaves regardless of age, representing 0.3−0.5% of that in stressed leaves. The ABA levels increased significantly in stressed leaves (L1 > L2> L3) and were positively correlated with age. Gibberellic acid 1 (GA1) levels increased in stressed leaves, especially in L2 and L3. IAA, zeatin (a cytokinin, CK), SA, and GA15, decreased or showed no significant changes upon osmotic stress, except for isopentenyladenine (a CK) levels, which increased at L3 (Fig. 2). Compared with the significantly increased ABA levels, *cis*- and *trans*-zeatin were significantly suppressed in mature leaves under osmotic stress (Fig. 2). This revealed that the dominant growth-promoting hormone shifted toward the stress hormone ABA, thereby initiating a series of stress-related responses for leaf adaptation to water deficit.

### Transcriptomic changes in different leaves under osmotic stress

**Fig. 3:**
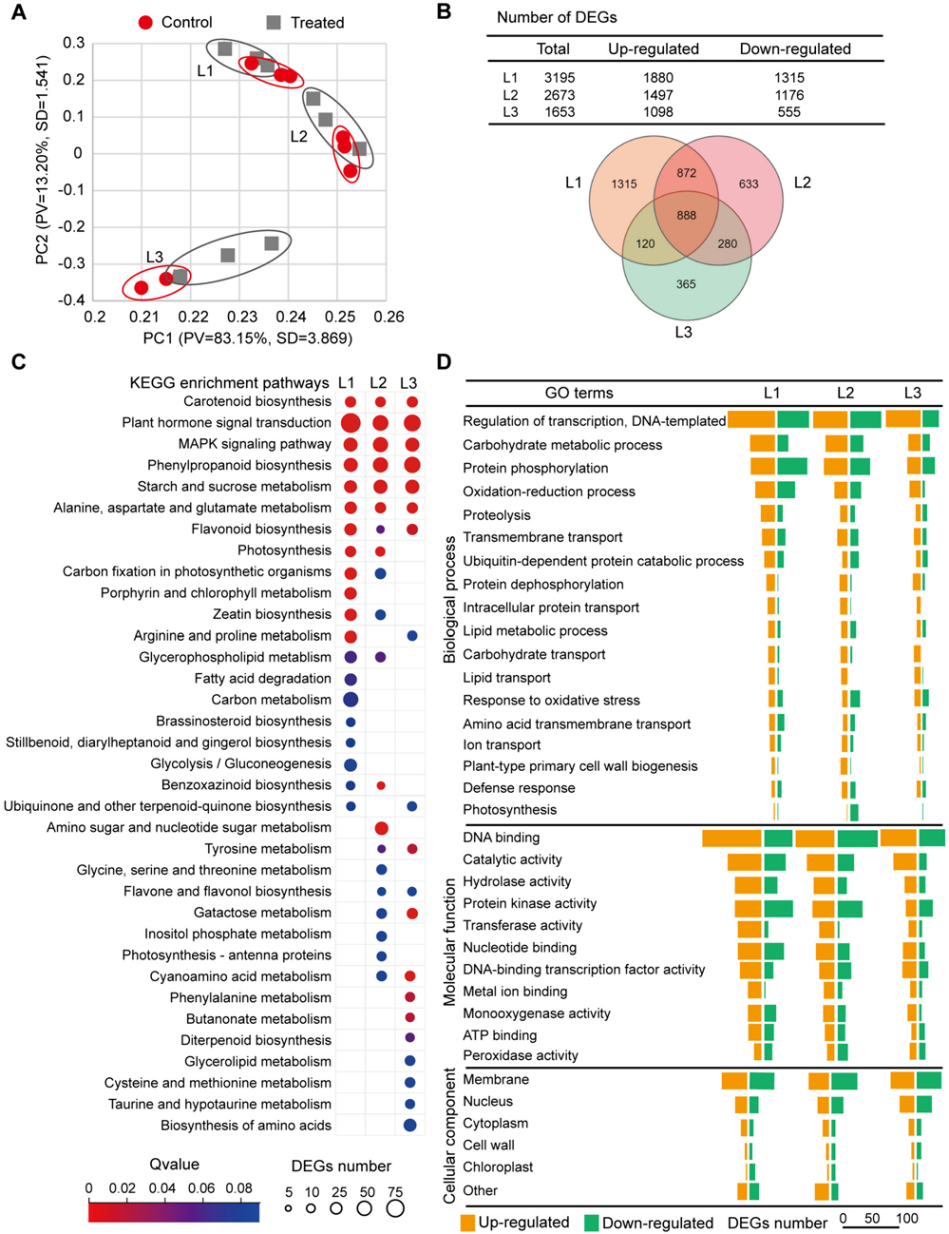
Summary on transcriptomic changes of maize leaves of three ages under osmotic stress. Ten-d-old seedlings were subjected to osmotic stress for 4 h, and each leaf was used for transcriptomic analysis. **A** PCA of transcriptome data sets of 18 different samples from L1, L2 and L3 (triplicate) under the control and stress conditions. TPM (transcripts per million) values were used to evaluate gene expression levels (*n*=3, principal components set at 5). The circles outline biological replicates for each leaf/treatment. The variation explained by the first two components and their scores (*Z*-values×eigenvector) are indicated on the axes. **B** Number of stress-responsive DEGs in leaves of three ages. *Upper*: Number of up-regulated/down-regulated DEGs. *Down*: Venn diagram of DEGs numbers. **C** Significant enriched KEGG pathways. **D** Significant enriched GO terms

Transcriptomic changes in leaves under osmotic stress were analyzed using RNA-seq. Quality checks confirmed the high quality of the clean reads in the three biological replicates (Supplementary Dataset 1). Reverse transcription quantitative PCR (RT-qPCR) revealed transcript levels of the 19 selected genes similar to those detected by sequencing (Supplementary Fig. 2). Tissue-level differences accounted for the largest variance (>83%) in the principal component analysis (PCA), whereas the control and treated samples were indistinguishable, especially for mature leaves (Fig. 3A).

We identified 3195, 2673, and 1653 stress-responsive DEGs in L1, L2, and L3, respectively (Fig. 3B; Supplementary Dataset 2). The Venn diagram (Fig. 3B) revealed 1315, 633, and 365 specific DEGs in L1, L2, and L3, respectively, which may be age dependent. Only 888 DEGs were common to all leaves and probably independent of age, the majority of which were upregulated.

These DEGs were mainly enriched in 35 KEGG pathways, seven of which were common to all leaves, including hormone signal transduction; the mitogen-activated protein kinase (MAPK) signaling pathway; phenylpropanoid biosynthesis; starch and sucrose metabolism; alanine, aspartate, and glutamate metabolism; flavonoid biosynthesis; and carotenoid biosynthesis (Fig. 3C). Each leaf included a distinct group of pathways under osmotic stress, such as porphyrin and Chl metabolism in L1; amino sugar and nucleotide sugar metabolism in L2; and phenylalanine and butanoate metabolism in L3 (Fig. 3C).

GO analysis identified 511, 463, and 332 terms in L1, L2, and L3, respectively (Supplementary Dataset 3). Stress-responsive DEGs were enriched in biological processes such as transcriptional regulation, carbohydrate metabolism, protein phosphorylation/dephosphorylation, oxidation/reduction process, proteolysis/ubiquitin-dependent protein catabolic process, oxidative stress, and defense response (Fig. 3D). The occurrence of upregulated DEGs related to proteolysis, ubiquitin-dependent protein catabolic processes, and transmembrane transport was higher in L1 than in L2 and L3. Molecular function terms such as DNA/nucleotide/ATP binding, catalytic activity, hydrolase/transferase/monooxygenase activity, metal ion binding, and transcription factor (TF) activity were linked to upregulated DEGs, thus favoring the adjustment of biological processes to osmotic stress (Fig. 3D). The cell components mainly included the membrane (43.05%), nucleus (21.80%), cytoplasm (8.84%), cell wall (5.64%), and chloroplasts (4.46%). The downregulated DEGs related to chloroplasts were obvious in mature leaves, but not in L3 leaves, consistent with the significantly decreased photosynthesis in mature leaves under osmotic stress (Fig. 1F).

Taken together, osmotic stress caused more extensive transcriptomic adjustments in L1, and multiple metabolic pathways and regulatory responses in leaves were affected, reflecting the physiological changes (Fig. 1), in line with the current understanding of plant stress responses.

## Proteomic changes in different leaves under osmotic stress

**Fig. 4:**
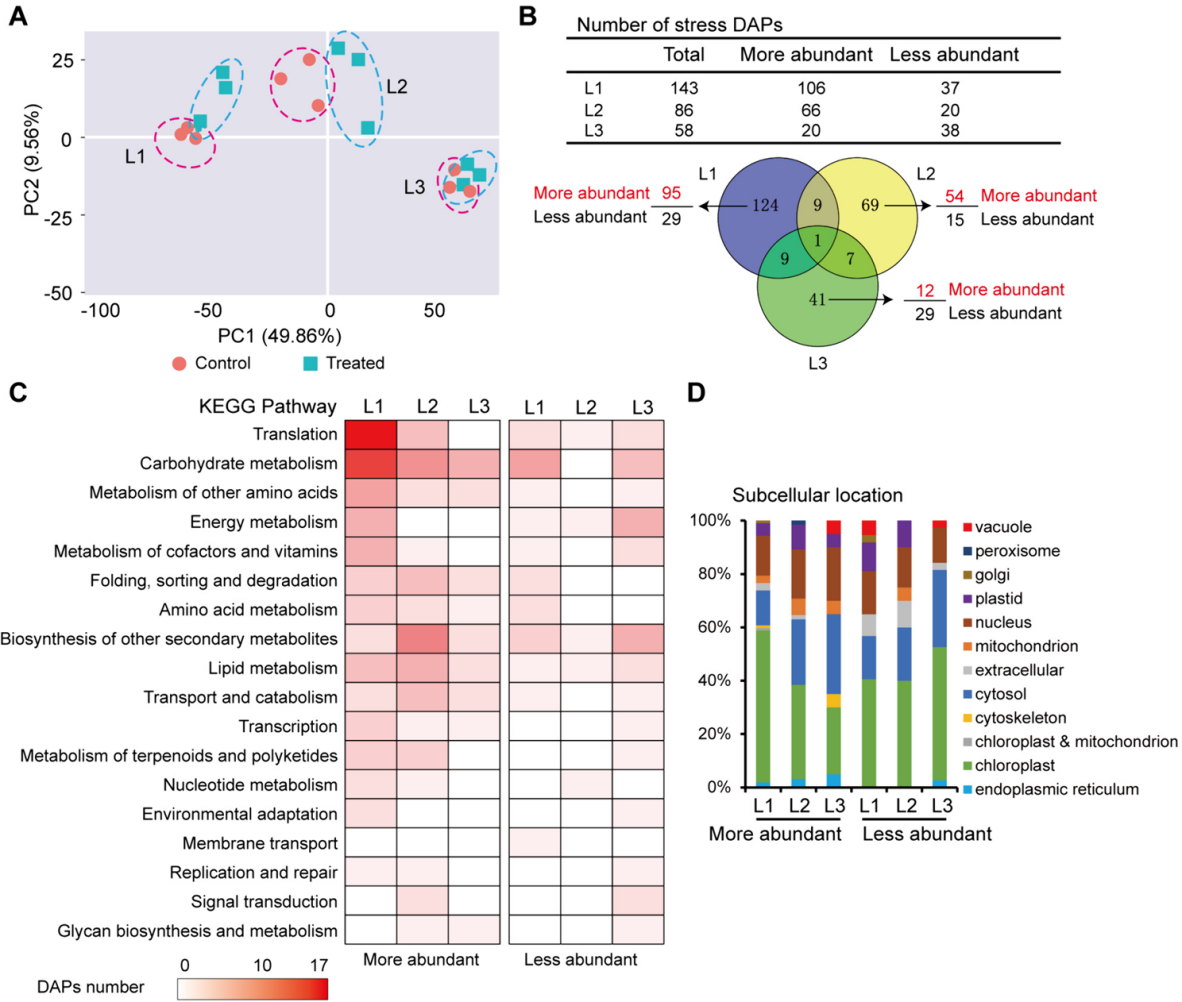
Summary on proteome changes in maize leaves of three ages under osmotic stress. Ten-d-old seedlings were subjected to osmotic stress for 4 h, and each leaf (triplicate) was used for proteomic analysis. **A** PCA of proteome data sets of 18 different samples from L1, L2 and L3 under the control and stress conditions. **B** Number of stress-induced DAPs. **C** KEGG pathway enrichment analysis of DAPs. **D** Subcellular locations of DAPs

We analyzed the proteomic responses of each leaf to osmotic stress. The identified 23676 peptides represented 6112 kinds of distinct proteins, 98.8% of which matched the corresponding genes detected by RNA-seq. Similar to the transcriptome data, tissue-level differences accounted for the largest variance (approximately 50%) of PCA in leaf proteomes (Fig. 4A), whereas the control and treated proteomes of L3 showed the least variance.

A paired comparison of the control and treated leaves led to the identification of 143, 86, and 58 stress-responsive DAPs in L1, L2, and L3, respectively (Supplementary Dataset 4; Fig. 4B). The DAPs number was only equivalent to 4.48% (L1), 3.22% (L2) and 3.51% (L3) of the corresponding DEGs number. This indicated that the proteomes could maintain a relatively steady state compared to the transcriptomes under short-term osmotic stress, especially for L3.

Most of these DAPs were age-related, with 124, 69, and 41 DAPs detected in L1, L2, and L3, respectively (Fig. 4B). More abundant DAPs represented a larger proportion of mature leaves. Only one common DAP (galactinol-sucrose galactosyltransferase) accumulated independently of age, with log_2_ fold-change (FC) values of 1.32, 1.26 and 1.6 in L1, L2, and L3, respectively. This enzyme transfers the galactosyl moiety of galactinol to sucrose, generating raffinose and inositol, which are involved in abiotic stress adaptation (Li et al. 2021).

The KEGG enrichment analysis revealed that the upregulated DAPs were enriched in 18 pathways (Fig. 4C). Translation, carbohydrate metabolism, amino acid metabolism, energy metabolism, secondary metabolite synthesis, protein folding, sorting, and degradation, transport and catabolism, lipid metabolism, and environmental adaptation were more significant in L1. Many pathways were common between the DAPs and DEGs. The predicted protein localization mainly included the chloroplasts, cytosol, and nuclei, with notable differences across the leaves (Fig. 4D).

Unexpectedly, an overwhelming majority of DAPs were not encoded by the corresponding DEGs (Supplementary Fig. 3), indicating a great inconsistency between protein abundance and transcription levels. The DAPs encoded by the DEGs included aquaporin TIP1-1, Protein EARLY RESPONSIVE TO DEHYDRATION 15, peroxidase, and the small ribosomal subunit protein uS2c. Although the abundance of 2-isopropylmalate synthase in L1 increased significantly, its expression was downregulated under osmotic stress. This enzyme participates in leucine biosynthesis, thus, a decrease in transcription but an increase in synthesis would maintain amino acid biosynthesis during leaf adaptation to water deficits.

## Metabolic pathways associated with osmotic stress tolerance in leaves

KEGG and GO analyses identified crucial DEGs and DAPs involved in the osmotic stress response in maize leaves. We further analyzed these DEGs and DAPs with respect to the enriched pathways. Similar to previous studies (Shi et al. 2013; Kanojia et al. 2020; Singh et al. 2022), we mainly focused on upregulated DEGs and more abundant DAPs, highlighting their important roles in abiotic stress resistance in plants (Zhang et al. 2022).

### Primary and energy metabolism

Photosynthesis and glycolysis provide essential compounds and energy for maintaining cellular homeostasis. Under osmotic stress, almost all DEGs linked to photosynthesis were downregulated, including *Chl a/b binding protein, PsbQ, PsaN, OHP, cytb*_*6*_*f, ATP synthase, and RuBisCO* (Supplementary Fig. 4A, B; Supplementary Dataset 5). These genes were suppressed the most in L1 and the least in L3, consistent with decreased photosynthesis. Only *PsbP*, *ferredoxin*, and *triose-phosphate isomerase* were upregulated in stressed leaves. Osmotic stress stimulated genes related to Chl degradation, such as *pheophorbide-a oxygenase* and *senescence-inducible chloroplast stay-green proteins*, but inhibited genes related to Chl synthesis, especially in L1 (Supplementary Fig. 4C). At the proteomic level, two Chl a/b-binding proteins and PsbP showed inconsistent changes in their corresponding gene levels, with PsbP being more abundant only in L3 (Supplementary Fig. 4A; Supplementary Dataset 5).

In photorespiration, the gene *glycolate oxidase* linked to peroxisomes was significantly downregulated in L1, but not in L3, whereas two genes encoding mitochondrial glycine cleavage system H protein2 (GDH2) were over-represented (Supplementary Fig. 4D), especially in mature leaves. Thus, the upregulated *GDH2* enhances the release of CO_2_ that can enter the Calvin cycle again, thereby protecting the photosynthetic apparatus under water deficit conditions.

**Fig. 5:**
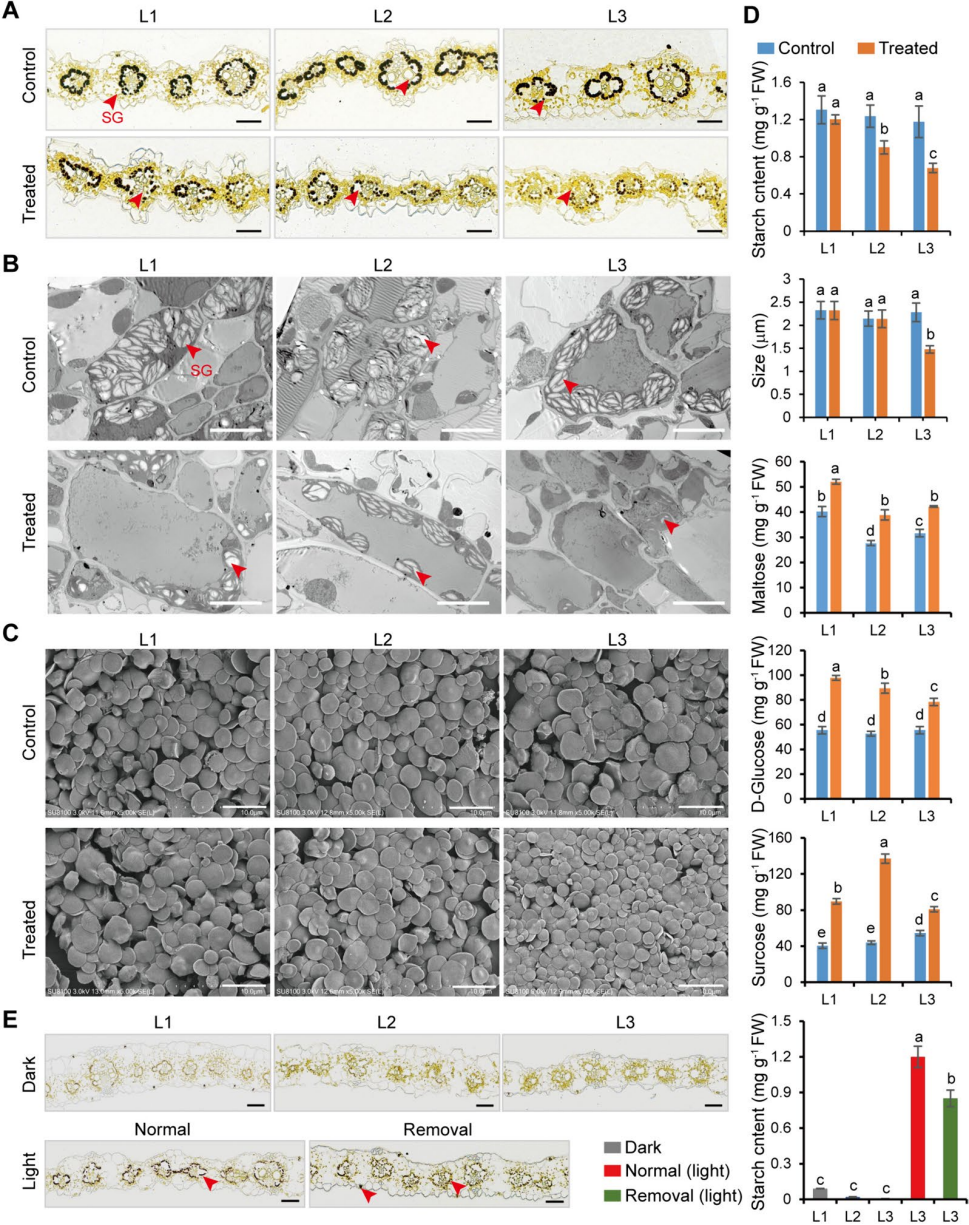
Response of starch granules (SG) and sugars metabolism to osmotic stress in leaves of three ages. Ten-d-old seedlings were subjected to osmotic stress for 4 h, and each leaf was used for microscopic analysis. **A** Iodine-stained cross section. Red arrows indicate SG. Scale bars, 50 μm. **B** Transmission electron microscopic images. Red arrows indicate SG. Scale bars, 5 μm. **C** Scanning electron microscopic images of purified SG from different leaves. Scale bars, 5 μm. **D** Determination of starch and sugar contents. SG size distribution was determined by measuring about 200 SG in triplicate. **E** Iodine-stained cross section of L3 after removing mature leaves. *Upper*: SG degradation in leaves under dark for 12 h. *Down*: SG accumulation in L3 under light for 12 h after removing mature leaves. Red arrows indicate SG. Scale bars, 50 μm. All experimental data were obtained from at least three biological replicates. Different letters above the columns indicate statistical significance in ANOVA (*P <* 0.05) using GraphPad Prism 8.0 software

Transitory starch is the main storage form of photosynthetic products; it accumulates in starch granules (SG) in chloroplasts during the day, and is hydrolyzed to soluble sugars at night (AbdElgawad et al. 2020). Microscopy observation indicated that SG existed only in vascular bundle sheath cells but not in mesophyll cells (Fig. 5A, B). Upon 4 h stress, SG degraded during light significantly more in L3 than in mature leaves, and the isolated SG (1.4 to 2.5 μm) from L3 were reduced in size compared with those from mature leaves (Fig. 5C). Meanwhile, maltose, glucose, and sucrose content increased in stressed leaves (Fig. 5D). After removing mature leaves, L3 accumulated adequate SG, comparable to the control (Fig. 5E).

Most genes linked to starch and sugar metabolism were upregulated in the stressed leaves (Supplementary Fig. 5A; Supplementary Dataset 6). Particularly, four *β-amylases* involved in starch degradation were upregulated, of which *ZmBAM8* had unusually high log_2_FC of 8.96, 8.63 and 6.84 in L1, L2 and L3, respectively. Most genes linked to sugar conversion and transport were also over-represented in stressed leaves, e.g., *sucrose-phosphate synthase*, three *UDP-glycosyltransferase* genes and *β-frucotofuranosidase* (Supplementary Fig. 5B; Supplementary Dataset 6)*.* Interestingly, *sucrose transporter 3* (*Suc3*) was only upregulated in L1. In Arabidopsis, sucrose export from the source tissues is mainly controlled by SUC2 activity (Tong et al. 2022). Hence, the upregulation of *Suc3* enhanced sucrose export from L1 to young leaves under osmotic stress. These results imply that SG degradation during the day plays a vital role in leaf stress adaptation, especially in L3, which differs from starch degradation at night to sustain plant growth and metabolism.

The glycolytic tricarboxylic acid (TCA) cycle plays a central role in energy metabolism. Key genes linked to glycolytic TCA were over-presented in stressed leaves, especially in L1, e.g., *pyruvate dehydrogenase kinase*, *NADH dehydrogenase*, *alternative oxidase AOX3 precursor*, *cyclic oxidative cytochrome C-2*, and *isocitrate lyase* (Supplementary Fig. 5C; Supplementary Dataset 6). Therefore, when chloroplast energy production declines under osmotic stress, mitochondrial and cytosolic energy pathways are enhanced to supply energy for plant stress adaptation.

### Differential defense response to osmotic stress via specific DEGs/DAPs

**Fig. 6:**
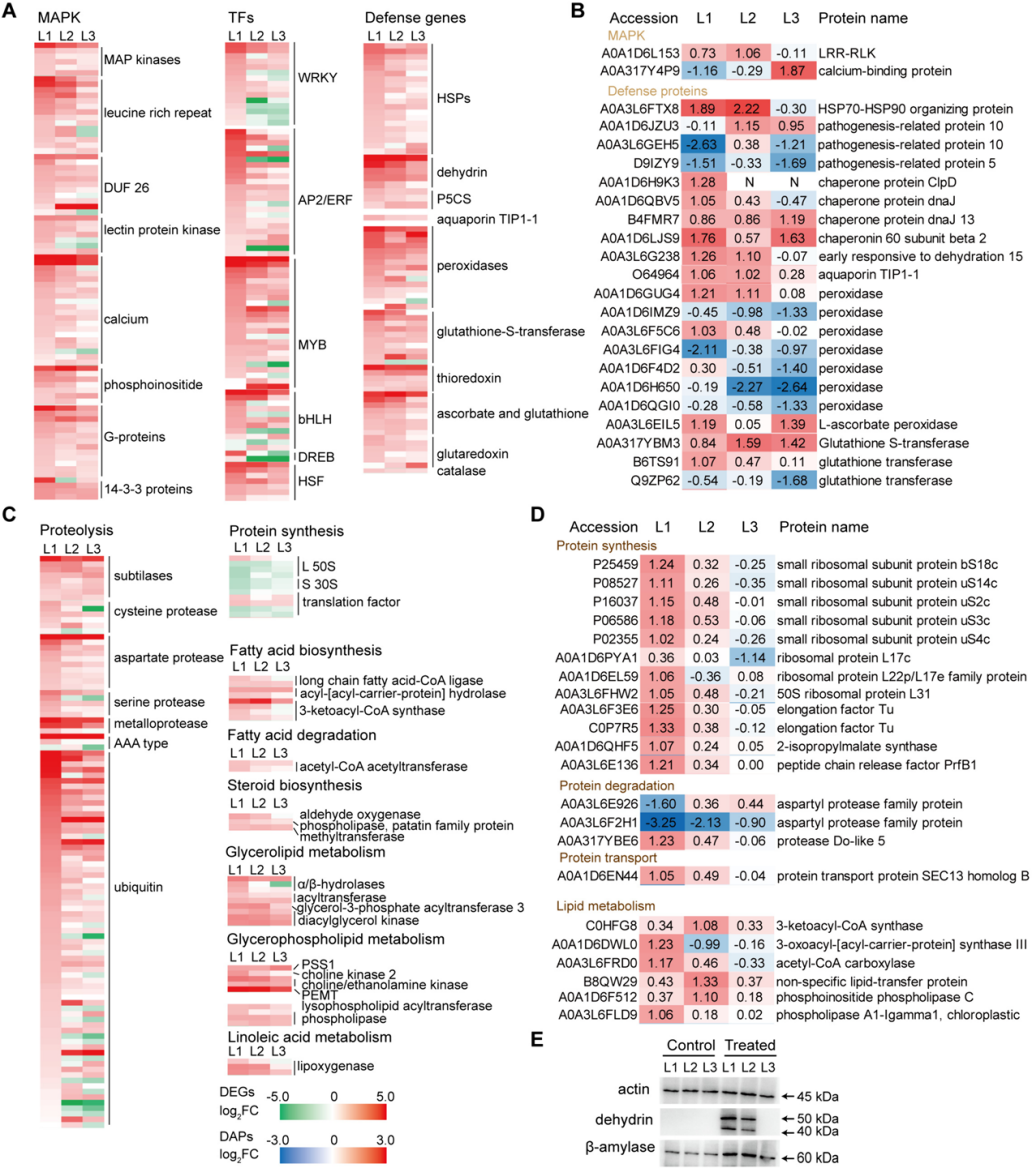
Key DEGs and DAPs involved in stress response of different maize leaves to osmotic stress. Ten-d-old seedlings were subjected to osmotic stress for 4 h. The osmotic stress induced differential changes in gene expression and protein accumulation in L1, L2 and L3 were highlighted. **A** DEGs related to mitogen-activated protein kinases (MAPKs), transcription factors (TFs) and defense response. **B** DAPs related to MAPKs, TFs and defense response. **C** DEGs related to synthesis and degradation of proteins and lipids. **D** DAPs related to synthesis and degradation of proteins and lipids. **E** Immunoblot detection of dehydrin and β-amylase. Equal protein loading was indicated by actin blot

Acute osmotic stress caused a reduction in leaf RWC (Fig. 1C), leading to dynamic alterations in gene expression and protein synthesis (Figs. 3 and 4). The heat map displayed significant changes in the selected DEGs and DAPs in individual leaves (Fig. 6), which were mainly involved in stress signaling, TF regulation, stress tolerance, ROS scavenging, and the synthesis and degradation of proteins and lipids.

A large number of MAPK cascade components were over-represented under osmotic stress, especially in L1 (Fig. 6A; Supplementary Dataset 7), including *MAPK, MAPK2, MAPK8, phospholipase C, cysteine-rich receptor-like kinase, leucine-rich repeat receptors, lectin receptor-like kinase, G-proteins, 14-3-3 proteins, and phosphoinositides*, which are known to encode key signal molecules that regulate plant development, metabolism, and stress response (Zhu 2016). Many genes associated with calcium sensing, binding, transport, and responsiveness were upregulated under osmotic stress, especially in L1 (Fig. 6A; Supplementary Dataset 7); encoding for plasma membrane calcium-transporting ATPase, Ca-binding protein, calmodulin, and chloroplastic GTP diphosphokinase. In particular, *calcium-transporting ATPase 5* was upregulated with high log_2_FC values of 5.05, 5.10 and 3.67 in L1, L2 and L3, respectively.

The upregulated MAPK and Ca^2+^ signaling pathways would trigger the activity of TFs, heat shock proteins (HSPs) and other stress proteins. The stress-responsive TFs genes include many *MYB*, *WRKY*, *APETALA2/ethylene-responsive factor* (AP2/*ERF)*, *bHLH*, *dehydration-responsive element-binding proteins* (*DREB*), and *heat shock factor* (*HSF*) (Supplementary Dataset 7), which are implicated in plant responses to multiple abiotic stresses (Zhu 2016; Zhang et al. 2022). Overall, more upregulated *WRKY, AP2/ERF, MYB, bHLH* and *HSF* genes were observed in L1 than in L2 and L3. The upregulated *bHLH* genes were eight, five, and four in L1, L2, and L3, respectively. The *bHLH93* is upregulated in mature leaves, and its homolog is involved in the differentiation of stomatal guard cells (Ohashi-Ito and Bergmann 2006).

Stress proteins such as HSPs and dehydrin play a vital role in conferring abiotic stress tolerance (Szlachtowska and Rurek 2023). Many genes encoding HSPs, chaperones, and dehydrin were upregulated more in L1 than in L2 and L3 (Fig. 6A). Among the 27 identified HSPs, 20 exhibited higher levels in L1. The chloroplast chaperone *ClpD* was upregulated in all stressed leaves; however, ClpD only increased in L1. Five different dehydrin genes were highly induced by osmotic stress, with the average log_2_FC of 5.48, 4.69, 3.17 in L1, L2, and L3, respectively. Immunoblotting revealed increased dehydrin levels in mature leaves under osmotic stress. Similarly, aquaporin TIP1-1 (Fig. 6B) increased in mature leaves, consistent with *TIP1-1* expression levels, likely permitting the rapid influx of water from old leaves into young leaves under water-deficient conditions.

A common strategy of plant responses to intracellular osmotic fluctuations is to accumulate osmolytes, such as proline. Delta-1-pyrroline-5-carboxylate synthase (P5CS) controlling the rate-limiting step in glutamate-derived proline biosynthesis. Three *P5CS* were upregulated in all stressed leaves (Fig. 6A; Supplementary Dataset 7). Meanwhile, a *MYB* that positively regulates proline synthesis and transport, was only upregulated in L1 (Supplementary Dataset 7).

Protein and lipid degradation pathways were enriched more evidently in L1 under osmotic stress (Fig. 3). Many genes involved in ubiquitin-mediated protein degradation, such as *cysteine protease*, *aspartate protease, ubiquitin-protein ligase,* and *RING-type E3 ubiquitin transferase* were upregulated in stressed leaves, with higher levels in L1 (Fig. 6C). Protease do-like 5, which is involved in chloroplast protein degradation, increased only in L1 (Fig. 6D). Genes linked to glycerolipid degradation (*α/β-hydrolases*) and fatty acid degradation (*acetyl-CoA acetyltransferase*) were upregulated in mature stressed leaves (Fig. 6C). Similarly, acetyl-CoA acetyltransferase was significantly upregulated in mature stressed leaves. Sphingolipids are fundamental components of the plant membrane system and are involved in multiple cellular and stress-response processes (Huby et al. 2020). The expression of sphingolipid biosynthesis-related genes, such as *ASC1-like protein 1* decreased in all stressed leaves, particularly in L1 (Fig. 6C; Supplementary Dataset 7).

### Hormones signal transduction

Hormone signal transduction was enriched in all leaves under osmotic stress (Fig. 3C). Numerous DEGs have been linked to the biosynthesis and signaling of auxins, ABA, zeatin, jasmonic acid (JA), and ethylene. Most of the DEGs (8 of 11) related to the ABA biosynthesis and response, were upregulated in all stressed leaves. The 9-cis-Epoxycarotenoid dioxygenase (NCED) is a key enzyme in ABA biosynthesis. HAV22 and ABF4 (ABRE binding factor 4) are involved in ABA-activated signaling. Four *NCED* and two *HAV22* genes, as well as *ABF4*, were upregulated by osmotic stress, with higher levels observed in mature leaves (Supplementary Fig. 6). Likewise, *MYB139* was upregulated in mature leaves (Supplementary Dataset 7), and its homolog *AtMYB44* repressed genes encoding phosphatase 2C in response to ABA (Jung et al. 2008). The expression levels of ABA-related genes positively correlated with leaf age, which was consistent with the actual ABA levels (Fig. 2C).

One SA-related gene was downregulated in all leaves, which was consistent with the reduced SA levels in all leaves (Fig 2; Supplementary Fig. 6). Most genes regulating JA biosynthesis, including *LOX1* (3), *LOX3* (1), *AOS* (1), and *OPR1* (2), were upregulated in L1, followed by L2, but were downregulated in L3, except for one *LOX1*, which was inconsistent with decreased JA levels in stressed leaves.

Ethylene is a well-known senescence-inducing hormone in plants (Rankenberg et al. 2021). Many TFs genes *AP2/ERF* were overrepresented under osmotic stress in L1 (17), followed by L2 (9), and L3 (7) (Supplementary Fig. 6). Three *ACO* genes (involved in ethylene biosynthesis) and two *ethylene-responsive protein* (*ERP*) genes were upregulated to comparable levels in all leaves (Supplementary Fig. 6). Without assaying ethylene levels, we could not compare the expression levels of ethylene-related genes.

Remarkably, genes related to hormones that regulate cell division and growth showed decreased expression in all stressed leaves, especially in L1 (Supplementary Fig. 6). Of the 15 auxin-related genes, the upregulated genes were more abundant in L2 (5) and L3 (5), whereas the downregulated genes were more abundant in L1 (6). Of the 11 zeatin-related genes, seven, two, and one were under-represented in L1, L2, and L3, respectively, consistent with zeatin changes (Fig. 2). GA-related genes were downregulated in all leaves, whereas *GA20 oxidase 1* was upregulated.

### Secondary metabolism

Secondary metabolites such as terpenoids, phenolics, and flavonoids play critical roles in plant adaptation to abiotic stress (Winkel-Shirley 2002). Multiple phenylpropanoid synthesis-related genes were upregulated in all the stressed leaves (Supplementary Fig. 7). In particular, many genes linked to the biosynthesis of terpenoids, betaine, lignin, and wax, were upregulated, especially in L1 (Supplementary Dataset 7). Genes encoding UDP-glucose 4-epimerase, UDP-glucose 6-dehydrogenase, UDP-glucuronate decarboxylase, and cellulose synthase which are associated with cell wall synthesis, were upregulated in mature leaves. In particular, *MYB20* which is involved in the regulation of secondary cell wall biogenesis, showed a high log_2_FC of 7.10, 7.54, 5.40 in L1, L2 and L3, respectively. By contrast, the genes encoding glycosyl hydrolase family 10 protein and mannan endo-1,4-β-mannosidase that are related to cell wall degradation were upregulated in mature leaves (Supplementary Fig. 7; Supplementary Dataset 7). Of the 15 genes linked to flavonoid biosynthesis, five were upregulated in L1. Five *MYB*s that regulate flavonoid and riboflavin biosynthesis were upregulated in all the stressed leaves. Moreover, the ZF-HD homeobox protein gene *ZHD15* which controls flavonoid biosynthesis, was highly expressed in L1 (log_2_FC 6.11) and L2 (log_2_FC 3.05), but slightly increased in L3 (log_2_FC 0.90).

## Discussion

**Fig. 7:**
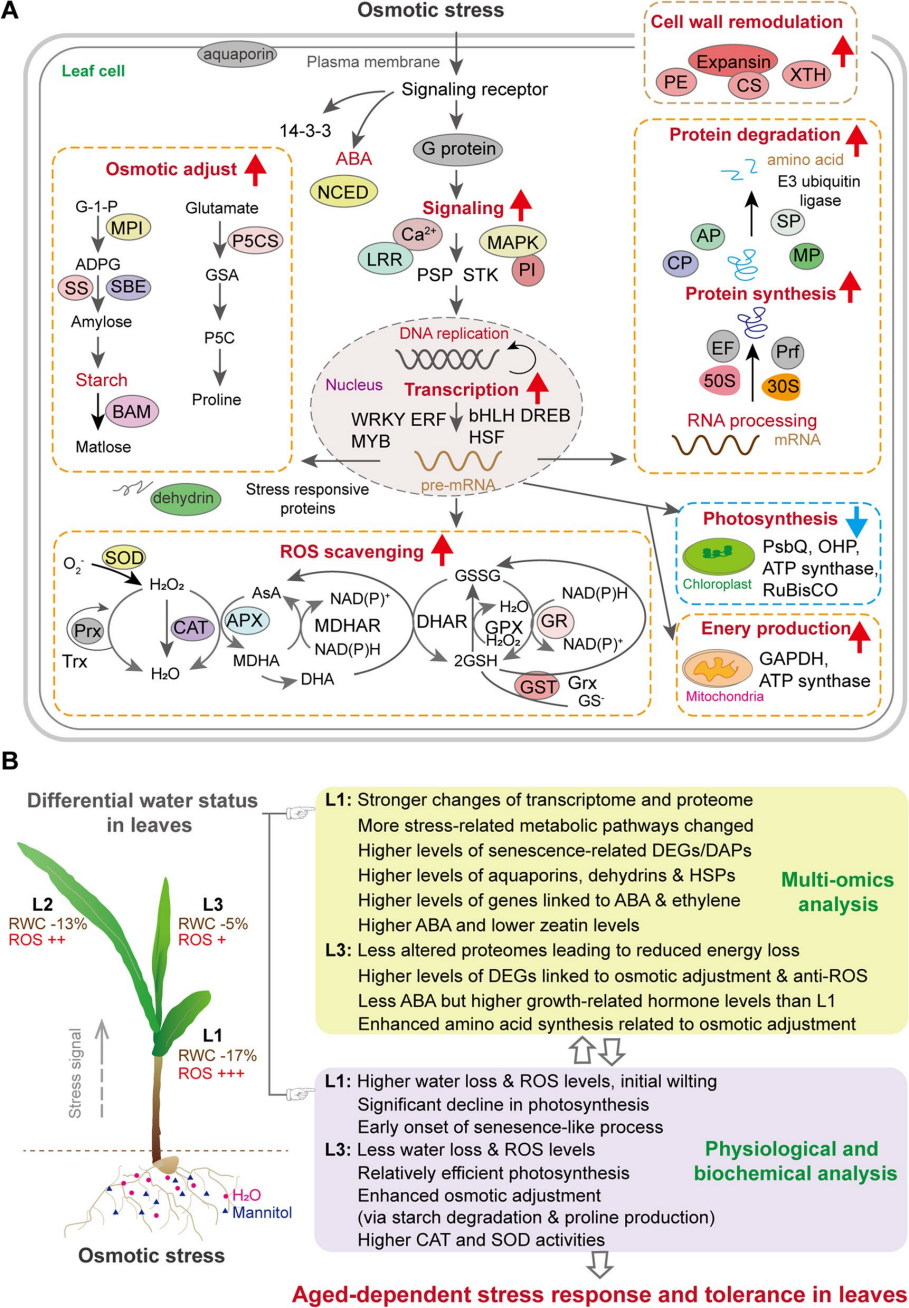
A Summary on the age-related changes in maize leaves in adaptation to osmotic stress. Ten-d-old seedlings were subjected to osmotic stress for 4 h. **A** Schematic map of the signaling and metabolic pathways in leaves in response to osmotic stress. **B** The osmotic stress induced differential responses in L1, L2 and L3. ABA, abscisic acid; AP, aspartate protease; APX, ascorbate peroxidase; BAM, β-amylase; CAT, catalase; Ca^2+^, calcium signals receptors; CP, cysteine protease; CS, cellulose synthase; EF, elongation factor Tu; GR, glutathione reductase; GSA, glutamate semi aldehyde; GST, glutathione S-transferase; LRR, leucine rich repeat; MAPK, mitogen-activated protein kinases; MP, metalloprotease; MPI, mannose-6-phosphate isomerase; NCED, nine-cis-epoxycarotenoid dioxygenase; PE, pectin esterase; PI, phosphoinositide; Prf, peptide chain release factor PrfB1; Prx, peroxidase; PSP: serine/threonine-protein phosphatase; P5C, pyrroline 5-carboxylate; P5CS, pyrroline 5-carboxylate synthase; SBE, starch-branching enzyme; SOD, superoxide dismutase; SP, serine protease; SS, starch synthase; STK, serine/threonine kinase; XTH, xyloglucan endotransglucosylase/hydrolase; 30S, 30S ribosomal protein; 50S, 50S ribosomal protein

Many studies have reported that old leaves are more susceptible to abiotic stress compared with young leaves (Wang et al. 2012; Kanojia et al. 2020). However, the mechanisms underlying this phenomenon in maize remain unclear. In this study, we aimed to gain a systematic understanding of age-related stress responses in maize leaves using a multi-omics approach. Mannitol has been used to induce osmotic stress (Nikonorova et al. 2018, Kalve et al. 2020) in the drought-resistant maize line C7-2t. As expected, old L1 was affected significantly more by osmotic stress relative to young L3, with greater levels and species alterations in DEGs, DAPs, and hormones (Figs. 1, 2, 3 and 4). These differences constitute the molecular basis of the age-related stress response mechanisms in maize leaves (Fig. 7).

*First, the age-related osmotic stress response was primarily determined by intracellular water status in individual leaves in the coordinated whole-plant defense responses.* Osmotic stress caused by drought is the primary signal that triggers a cascade of complex secondary reactions and metabolic regulatory disorders in plants (Zhang et al. 2022). When exposed to short-term osmotic stress in the roots, maize plants respond with stomatal closure, to mitigate water loss via transpiration. The water deficit status in individual leaves varied with age, and the RWC reduction was significantly higher in L1 than in L3 (Fig. 1C). Leaf RWC reflects the physiological consequences of cellular water deficit (Nevo and Chen 2010). Hence, L3 exhibited a more effective water conservation strategy than L1 in the coordinated whole-plant stress response, owing to the effects of both water potential and osmotic adjustment (Fig. 7).

The effect of osmotic stress on maize leaves of different ages evidently differs from that of other abiotic stresses, such as heat and cold. For instance, heat treatment generates a similar temperature effect on leaves of different ages in maize (Karim et al. 1999) and Arabidopsis (Xiang and Rathinasabapathi 2022), whereas osmotic stress imposed on maize roots causes differential water deficits across leaves. In particular, more DEGs were detected in young than in old leaves of Arabidopsis under heat stress (Xiang and Rathinasabapathi 2022), whereas in the current work we observed an opposite trend under osmotic stress (Fig. 3). The differential tolerance of young and old leaves to osmotic stress cannot be explained simply by the loss of stress tolerance with age, as observed in Arabidopsis (Kanojia et al. 2020). We compared RWC in the three leaves of the same maize plants, whereas Kanojia et al. (2020) used 10-, 15-, and 20-d-old Arabidopsis seedlings for six days of drought stress and compared RWC among the first rosette leaves from different plants of three ages. Maize leaves used here were not aging, and even 8-d-old L1 had an undetectable ABA, with similar Chl fluorescence to that of 5-d-old L2 under control conditions (Figs. 1C and 2). No visible programmed cell death was detected across leaves (Supplementary Fig. 8), whereas the first rosette leaves in Arabidopsis were progressively aging.

The osmoprotectant proline showed the greatest increase in L3 compared to mature leaves (Fig. 1G). As a result, osmotic adjustment may be more active in L3 to maintain cellular water content. Aquaporin is required for rapid transcellular water flow through living cells (Chaumont and Tyerman 2014). Aquaporin significantly increased upon osmotic stress in L1 at the transcript and protein levels (Fig. 6), and a *MYB* regulating proline transport and *sucrose transporter 3* (*Suc3*), was upregulated only in L1, probably promoting water, proline, and sucrose export from L1 to young leaves under osmotic stress. It appears that maize plants tend to protect young leaves by extracting water and nutrients from older leaves under osmotic stress, but no relevant experiments have yet provided support for this conclusion (Rabas and Martin 2003). Based on the multi-omics data obtained here, we propose a schematic map of the signaling and metabolic pathways in maize leaves in response to osmotic stress, highlighting the key molecular responses occurring in L1 (Fig. 7). Taken together, leaf age determined the extent of the intracellular water deficit under short-term osmotic stress in our experimental conditions, thereby causing significant changes in transcriptomes, proteomes, and hormone levels across leaves.

*Second, the adjustment of transcriptome and proteome in old leaves to osmotic stress was higher than that in young leaves.* Leaf initiation and development involve cell proliferation and expansion, which mediate differential gene expression linked to specific cellular processes and signaling pathways of hormones, especially auxin, CK, and GA (Rankenberg et al. 2021). Leaf age-related stress responses are closely related to the developmental stages. Leaf age also correlates with the import of proteins into chloroplasts (Teng et al. 2012) and photosynthetic activity (Sperdouli et al. 2021). PCA revealed that differential clustering was more evident by age than by stress (Figs. 3A and 4A); that is, transcriptomic and proteomic adjustments to osmotic stress were mostly dependent on leaf age. Under the combined effects of leaf age and osmotic stress, the transcriptomes fluctuated more than the proteomes, especially in L1 (Figs. 3B and 4B). The less-altered proteomes in L3 may reduce the energy consumption of non-essential metabolic activities in cells, thereby facilitating L3 adaptation to osmotic stress.

The occurrence of senescence-like cellular events were higher in L1 than in L3 under osmotic stress, such as proteolysis, ubiquitin-mediated protein degradation, and lipid degradation (Figs. 3D, 4C and 7). Many senescence-associated genes, such as *AP2/ERF, SAG12, SAG20, DIN1*, *senescence regulators, luminal-binding protein*, and *senescence-inducible chloroplast stay-green proteins* (*SGR1, SGR2;* Park et al. 2007) were overexpressed, especially in L1 (Supplementary Fig. 4C, Fig. 6). The *SAG12* and *SAG20* homologs are upregulated during the early stages of leaf senescence (Kanojia et al. 2020, Xu and Xue 2019). Luminal-binding protein (an HSP70) recognizes misfolded proteins in the lumen of the endoplasmic reticulum, and regulates protein degradation via autophagy (Valente et al. 2009). Compared with L3, the severe water deficit in L1 caused an early onset of leaf senescence, subsequently resulting in a significant enrichment for stress proteins (e.g., HSPs, chaperones, dehydrin, and aquaporin), and those involved in nutrient remobilization at the transcriptome and proteome levels. L3 is not responsive toward senescence-inducing factors, probably to protect younger leaves (Rankenberg et al. 2021). The occurrence of senescence-like cellular processes in L1 may enhance the nutrition released from protein degradation and export into young leaves, which is beneficial for plant survival under osmotic stress. Notably, a mild water deficit in L3 did not cause obvious protein degradation or denaturation, and the accumulation of stress proteins was not as significant as that in L1, except for dehydrin (Fig. 6A).

The antioxidant systems act differently in leaves of different ages. L3 exhibited a greater increase in CAT and SOD activities, but mature leaves had high peroxidase (POD) activity at the protein and transcript levels (Figs. 1G and 6A, B) to protect themselves from oxidative damage. These POD are localized in the cell wall or extracellular region and function in the removal of ROS, wall biosynthesis, and stress responses in mature maize tissues (Niu et al. 2019b, 2020).

DAPs and DEGs exhibited weak consistency of 21.7% in L1, 17.4% in L2, and 15.5% in L3 (Supplementary Fig. 3). The consistency ranges from 27% to 40% (Muers et al. 2011; Vogel et al. 2012; Walley et al. 2016; Jia et al. 2018), depending on the species, cell type, and developmental stage. For example, it is quite low in immature tomato fruits (Osorio et al. 2011) and in the ‘sink-source’ transition zone of maize leaves (Ponnala et al. 2014). Other factors that influence this correlation include translational control, protein degradation, modifications, and complex formation (Vogel et al. 2012). Weak consistency between transcripts and proteins implies active reprogramming of protein translation under osmotic stress.

*Third, metabolic activities and stress signaling pathways were significantly influenced by osmotic stress in an age-related manner.* Photosynthesis is sensitive to abiotic stress (AbdElgawad et al. 2020). Compared with L1, L3 maintained relatively high levels of photosynthetic proteins and activity under osmotic stress (Supplementary Fig. 4A), as observed in heat-stressed maize leaves (Karim et al. 1999). In Arabidopsis, young leaves are less vulnerable to drought than older leaves, largely because young leaves maintain an almost normal PSII function to avoid photooxidative damage (Sperdouli et al. 2021). Under osmotic stress, the decreased photosynthesis (Fig. 1F, Supplementary Fig. 4A, B) was partly compensated for by the increased glycolytic TCA (Supplementary Fig. 5) to support the stress response and metabolic activities, such as during leaf senescence (Luo et al. 2020). Thus, maintaining sufficient photosynthetic activity in L3 enhanced stress tolerance. In addition, differential secondary metabolism was observed in all the stressed leaves.

Plants accumulate specific primary metabolites as an adaptive response to abiotic stress (Urrutia et al. 2021; Zhang et al. 2022). Starch and sucrose metabolisms were implicated in the leaf response to osmotic stress (Figs. 3C, D and 4C). Transitory starch is a major determinant of plant growth and responses to abiotic stresses, including salt, drought, and cold (Thalmann and Santelia 2017; Dong and Beckles 2019; AbdElgawad et al. 2020). Arabidopsis leaves accumulated transitory starch in the chloroplasts of mesophyll cells (Crumpton-Taylor et al. 2012), whereas maize leaves accumulated SG only in the chloroplasts of vascular bundle sheath cells (Supplementary Fig. 5A, B). Short-term osmotic stress cause rapid starch decreased in Arabidopsis leaves kept under dark conditions, whereas long-term stress significantly increases starch content (Liu et al. 2019). We found that transitory starch levels declined rapidly under osmotic stress, even in daylight, especially in L3 (Supplementary Fig. 5A–C), which was accompanied by an increase in soluble sugars (Supplementary Fig. 5D). Young leaves did not rely on mature leaves to produce transitory starch (Supplementary Fig. 5E), which contributed to the maintenance of photosynthesis in maize leaves under drought (AbdElgawad et al. 2020). In addition, three MYB genes were more suppressed by osmotic stress in L3 and L2 than in L1, and their homolog OsMYB30 suppresses *β-amylase* expression in rice (Lv et al. 2017). Thus, transitory starch plays a vital role in the osmotic stress response of leaves (especially L3) through rapid degradation, to redirect carbon for stress adaptation.

Under osmotic stress, the MAPK and hormone signaling pathways were significantly activated in mature leaves, causing stronger stress responses and senescence-like changes in L1 (Figs. 2, 6A and 7; Supplementary Fig. 6). ABA and ethylene are senescence-activating hormones, whereas CK has the opposite effect (Rankenberg et al. 2021). CK-related genes differ significantly in their transcript numbers and levels between young and old leaf in tomato (Shi et al. 2013). Here, ABA content and ABA-responsive transcripts were the highest in L1, but two zeatins and their related transcripts declined drastically in mature leaves (Fig. 2; Supplementary Fig. 6). Many genes involved in ethylene biosynthesis and its responses were upregulated, especially in mature leaves (Supplementary Fig. 6). Evidently ABA, ethylene, and CK mediate stress-induced senescence in maize in an age-dependent manner.

In conclusion, the multi-omics data generated here revealed common and specific stress responses in maize leaves under the combined effects of age and stress. The extent of water deficit in individual leaves varied significantly under short-term osmotic stress, thereby causing age-dependent differential changes in transcriptomes, proteomes, and hormones. Many upregulated DEGs and DAPs have the potential to improve stress tolerance in maize. Overall, our study provides insights that may further deepen our understanding of the age-related stress response mechanisms in plants.

## Materials and methods

### Plant materials, growth conditions and phenotyping

Maize drought-tolerant line C7-2t, generated by irradiation mutagenesis of the C7-2 inbred line (Zhang et al. 2020), was used here. Plants were grown in 1/4 Hoagland medium under controlled conditions (27°C, 65% relative humidity, 16 h light: 8 h dark photoperiod, and 150 μmol m^-2^s^-1^). At 10 d after germination, half of the seedlings were subjected to 0.3 M mannitol stress (in 1/4 Hoagland medium) for 4 h under 5 h light; the other half served as the control. Afterwards, the fully expanded leaf (L1), the newly-developed leaf (L2), and the developing leaf (L3), were harvested separately from the control and stressed plants. Each sample consisted of three biological replicates. In total, 18 samples from three age groups and two conditions were compared pairwise between the control and stressed samples to determine significant FCs in transcripts, proteins, and hormones. Moreover, mature L1 and L2 were removed and only L3 was retained in the seedlings to evaluate its effects on starch metabolism.

The RWC, as well as proline, and MDA contents were determined as previously described (Zhang et al. 2020). Leaf water loss was evaluated using detached leaves (Liu et al. 2023). The H_2_O_2_ levels and GSH, SOD, POD, and CAT activities were assayed as described previously (Yang et al. 2021). Proteins were extracted using a phenol-based method (Wu et al. 2014). Chl, protein, starch, and soluble sugars were quantified as previously described (Niu et al. 2019a). Chl fluorescence and the maximum photochemical efficiency of PSII (Fv/Fm) were measured using a multicolor fluorescence device after the seedlings were dark-adapted for 15 min. Stomatal characteristics were observed as described (Liu et al. 2023). Dehydrin and β-amylase were detected with specific antiserums (AS07 206A, AS15 2895, Agrisera) by immunoblotting leaf protein (Niu et al. 2019a), using anti-actin antibodies to confirm equal protein loading. All experiments were performed three times independently.

### Light and electron microscopy

For light microscopy, leaf tissue was fixed, dehydrated, embedded, and sectioned as previously described (Du et al. 2019). The sections were stained with safranin-fast green or iodine and observed under a light microscope. The SG were isolated from the leaves according to Niu (*et al.* 2019a), and observed using scanning electron microscopy. For transmission electron microscopy, the leaves were transversely cut using an ultramicrotome. Sections were mounted onto aluminum electron microscope stubs, coated with a thin layer of gold using a vacuum sputter coater, and observed using an HT7700 system (Hitachi, Japan). Images were processed using the CaseViewer software (v. 3.3.6).

### Hormone profiling by targeted metabolomics

Leaf tissue (50 mg) was ground in 1.0 ml of 50% acetonitrile (plus internal standard) in a grinding tube with magnetic beads (50 Hz, 5 min), followed by rotary extraction (1 h, 4°C). After centrifugation (12000*g*, 15 min, 4°C), the supernatant was filtrated, freeze-dried and redissolved in 50 μl of 50% acetonitrile. Liquid chromatography-tandem mass spectrometry (LC-MS/MS) was performed using a QTRAP 6500+ system (SCIEX) at the BGI (Shenzhen, China). Samples (10 μl) were injected into the HSS T3 Column (Waters, USA) and eluted using eluent A (0.02% formic acid) and eluent B (0.02% formic acid + acetonitrile). The solvent gradient was set as 0–3 min 2% B, 3–15 min 80% B, 15–18 min 98% B, 18–20 min 2% B. Ion source parameters were 500 °C, 5000 V, curtain gas 30 psi, gas I 40 psi, gas II 40 psi, collision gas 8 psi for positive mode, and 450°C, –4500 V, 35, 55, 45, and 9 psi for the negative mode. Multiple reaction monitoring was performed for metabolite identification and quantification using the Novogene database.

### Transcriptome profiling by RNA-Seq

Total RNA was isolated from 100 mg of leaf tissue using an RNA extraction kit (Qiagen, Hilden, Germany). The mRNA was enriched by polyA selection and sequenced using the BGIseq500 system at BGI (Shenzhen, China). Raw reads were filtered using the SOAPnuke software to remove adapters and low-quality reads. HISAT2 was used to align clean reads to the maize reference genome (GCF_000005005.2_B73_RefGen_v4) (Kim et al. 2015). DEGs with |log_2_FC| ≥ 1 (*q* ≤ 0.05) were identified by DESeq2 (Love et al. 2014). GO and KEGG enrichment analyses for DEGs were used to identify stress-related metabolic pathways (Liu et al. 2023). The sequence data were deposited in the European Nucleotide Archive (accession no. E-MTAB-11331). The expression levels of the 19 selected transcripts were verified using RT-qPCR (primers listed in Supplementary Table 1), with *ZmUBI* as an internal control.

### Proteome profiling by data-independent acquisition (DIA) proteomics

Leaf proteins were extracted using a phenol-based method (Wu et al. 2014). After reduction and alkylation, 100 μg proteins were digested overnight at 37 °C with 0.5 µg sequencing-grade trypsin (Niu et al. 2019b). The resultant peptides were desalted, vacuum-dried, redissolved in mobile phase A (5% acetonitrile, pH 9.8), and separated on a Gemini high-pH C18 column using a Shimadzu LC-20AB HPLC system. Next, the peptides were reconstituted in mobile phase A (2% acetonitrile and 0.1% formic acid) and separated using a Thermo UltiMate 3000 UHPLC. After enrichment and desalting, the samples were placed on a tandem self-packed C18 column and separated using mobile phase B (98% acetonitrile and 0.1% formic acid). The separated peptides were ionized using a nanoESI source and passed through an Oritrap Exploris 480 tandem mass spectrometer (Thermo Fisher Scientific) for data-dependent acquisition (DDA) mode detection. The DDA data were identified using the Andromeda search engine against UniProtKB (January 27, 2022). DAPs were identified based on |log_2_FC| ≥ 1 (*q* ≤ 0.05) for significant difference by MSstats in R (Choi et al. 2014). Finally, GO and KEGG enrichment analyses of DAPs were used to identify stress-related metabolic pathways. The proteome data were deposited in the ProteomeX Change Consortium (accession no. PXD037099).

### Statistical analyses

Spearman correlation coefficient was computed using the ‘test. Corr’ in R package; principal component analysis (PCA) and analysis of variance (ANOVA) were calculated using ‘metaX’ in R package (Wen et al. 2017). Each experiment was performed in at least three biological replicates. To determine statistical significance of the raw data, an ANOVA (*P <* 0.05) test or Student's *t*-test (*P* < 0.05) were performed using GraphPad Prism 8.0 software.

### Supplementary Information

**Additional file 1:** **Supplementary Fig. 1.** Comparison of stomata characteristics of different leaves. The abaxial surfaces of leaves in 10-d-old maize seedlings were observed under a microscope. **Supplementary Fig. 2.** Reverse transcription quantitative-PCR (RT-qPCR) validation of expression changes of 19 DEGs in different maize leaves. Ten-d-old seedlings were subjected to osmotic stress for 4 h, and each leaf was used for RNA extraction and PCR analysis (triplicate). Mean relative expression levels of DEGs were normalized to a value of 1.0 in L1 under control condition with *ZmUBI* as a reference gene. Error bars indicated the SE values of three biological replicates. Asterisks show significant difference in expression changes as assessed by Student's *t*-test (* *P* < 0.05, ** *P* < 0.01, *** *P* < 0.001). **Supplementary Fig. 3.** Correlation analyses of transcriptomic and proteomic data sets in different maize leaves under control and osmotic stress conditions. Ten-d-old seedlings were subjected to osmotic stress for 4 h, and each leaf was used for transcriptomic and proteomic analyses. A Number correlations between proteins and mRNAs and between DAPs and DEGs. B Expression correlations between proteins and mRNA and between DAPs and DEGs. NDEGs, no differences in gene expression; NDAPs, no differences in protein expression. C Classification distribution map of term associated genes significantly enriched in GO and KEGG pathway. The numbers in parentheses represent the number of proteins associated with the term, and the color of the heat map represents the proportion of the protein associated with the GO and KEGG terms. **Supplementary Fig. 4.** DEGs and DAPs involved in photosynthesis, photorespiration and Chl metabolism in different-age maize leaves under osmotic stress. Ten-d-old seedlings were subjected to osmotic stress for 4 h, and each leaf was used for transcriptomic and proteomic analyses. A Light reaction. B Calvin cycle. C DEGs involved in Chl biosynthesis and degradation. D Photorespiration. **Supplementary Fig. 5.** DEGs and DAPs involved in starch and sugar metabolism and glycolysis in different maize leaves under osmotic stress. Ten-d-old seedlings were subjected to osmotic stress for 4 h, and each leaf was used for analyses. A starch metabolism. B Sugars metabolism. CGlycolysis-TCA. **Supplementary Fig. 6.** The transcript levels of DEGs related to phytohormones in different maize leaves under osmotic stress. Ten-d-old seedlings were subjected to osmotic stress for 4 h, and each leaf was used for analyses. Abbreviations: 2OGDD, 2-oxoglutarate-dependent dioxygenase; ACO, 1-aminocyclopropane-1-carboxylate oxidase; ABF4, ABRE binding factor 4; AUX1, auxin transporter 1; AOS, allene oxide synthase; CKO, CK oxidase; CKX, CK oxidase/dehydrogenase; ERP, ethylene-responsive protein; GA20OX1, GA20 oxidase; GA2OX1, GA2 oxidase 1; GEM, GL2 expression modulator; GH3.17, IAA-amido synthetases; GRAM, GRAM domain-containing protein; GT, glycosyltransferase; HVA22, abscisic acid-responsive HVA22 family protein; ILR1, IAA-leucine resistant 1; LOX, Lipoxygenase; NCED, nine-cis-epoxycarotenoid dioxygenase; JMT, jasmonic acid carboxyl methyltransferase; OPR1, 12-oxophytodienoate reductase 1; PIN, PIN-formed; SUAR, auxin-responsive SAUR family protein; SCL, scarecrow-like transcription factor. Data represent means ± SD of three biological replicates. Significant differences in expression levels are indicated with different letters (*P* < 0.05, ANOVA). **Supplementary Fig. 7.** DEGs related to secondary metabolism in different maize leaves under osmotic stress. Ten-d-old seedlings were subjected to osmotic stress for 4 h, and each leaf was used for analyses. A DEGs related to wall synthesis and degradation. B DEGs related to various secondary metabolites. C DAPs related to secondary metabolism. Abbreviations: DBAT, 10-deacetylbaccatin III 10-O-acetyltransferase; DXR, 1-deoxy-D-xylulose-5-phosphate reductoisomerase; HMGR, 3-hydroxy-3-methylglutaryl-coenzyme A reductase; XTH, xyloglucan endotransglucosylase/hydrolase. **Supplementary Fig. 8.** Programmed cell death (PCD) detection in different maize leaves under osmotic stress. Ten-d-old seedlings were subjected to osmotic stress for 4 h, and each leaf was used for analyses. PCD was detected using Fluorescein (FITC) Tunel Cell Apoptosis Detection Kit (Servicebio, Wuhan, China). The blue signal represents staining with propidium iodide (PI), green signal represents TUNEL-positive nuclei of dead cells due to PCD. The images were detected with a Zeiss LSM880 confocal laser scanning microscope. Scale bars, 100 mm.

**Additional file 2:** **Supplementary Table 1.** Primers used in this study.

**Additional file 3:** **Supplementary Dataset 1.** Gene sequence assembly, quality check, alternative splicing, and expression levels of maize leaf transcriptomes.

**Additional file 4:** **Supplementary Dataset 2.** DEGs in maize leaves of different ages under osmotic stress. **Supplementary Dataset 3.** GO terms of DEGs in maize leaves of different ages under osmotic stress. **Supplementary Dataset 4.** DAPs in maize leaves of different ages under osmotic stress. **Supplementary Dataset 5.** DEGs related to photosynthesis. **Supplementary Dataset 6.** DEGs related to carbohydrate and glycolytic-TCA metabolism. **Supplementary Dataset 7.** DEGs related to stress response.

## Data Availability

All data included in this study are available as Supporting Information. RNA-Seq data were deposited in the European Nucleotide Archive (accession No. E-MTAB-11331, https://www.ebi.ac.uk/ena/browser/text-search?query=E-MTAB-11331). Proteome data were deposited in the ProteomeXchange Consortium (accession No. PXD037099, http://www.ebi.ac.uk/pride, Username: reviewer_pxd037099@ebi.ac.uk; Password: joOfUdnI).

## Abbreviations

ABA: Abscisic acid
ABF4: ABRE binding factor 4
AP2/ERF: APETALA2/ethylene-responsive factor
CAT: Catalase
CK: Cytokinin
DAPs: Differentially abundant proteins
DDA: Data-dependent acquisition
DEGs: Differentially expressed genes
DIA: Data-independent acquisition
DREB: Dehydration-responsive element-binding proteins
ERP: Ethylene-responsive protein
FC: Fold-change
GA1: Gibberellic acid 1
GDH2: Glycine cleavage system H protein2
GSH: Glutathione
HSF: Heat shock factor
HSPs: Heat shock proteins
JA: Jasmonic acid
LC-MS/MS: Liquid chromatography-tandem mass spectrometry
MAPK: Mitogen-activated protein kinase
MDA: Malondialdehyde
NCED: 9-cis-epoxycarotenoid dioxygenase
PCA: Principal component analysis
POD: Peroxidase
P5CS: Delta-1-pyrroline-5-carboxylate synthase
ROS: Reactive oxygen species
RT-qPCR: Reverse transcription quantitative PCR
RWC: Relative water content
SA: Salicylic acid
SG: Starch granules
SOD: Superoxide dismutase
Suc3: Sucrose transporter 3
TCA: Tricarboxylic acid
