# Supplementary material for: Maize multi-omics reveal leaf water status controlling of differential transcriptomes, proteomes and hormones as mechanisms of age-dependent osmotic stress response in leaves
Source: Stress Biol. 2024 Mar 18;4(1):19. doi: 10.1007/s44154-024-00159-9 (PMC10948690; doi:10.1007/s44154-024-00159-9)
Supplement: Supplementary file 1 — Additional file 1: Supplementary Fig. 1. Comparison of stomata characteristics of different leaves. The abaxial surfaces of leaves in 10-d-old maize seedlings were observed under a microscope. Supplementary Fig. 2. Reverse transcription quantitative-PCR (RT-qPCR) validation of expression changes of 19 DEGs in different maize leaves. Ten-d-old seedlings were subjected to osmotic stress for 4 h, and each leaf was used for RNA extraction and PCR analysis (triplicate). Mean relative expression levels of DEGs were normalized to a value of 1.0 in L1 under control condition with ZmUBI as a reference gene. Error bars indicated the SE values of three biological replicates. Asterisks show significant difference in expression changes as assessed by Student's t-test (* P < 0.05, ** P < 0.01, *** P < 0.001). Supplementary Fig. 3. Correlation analyses of transcriptomic and proteomic data sets in different maize leaves under control and osmotic stress conditions. Ten-d-old seedlings were subjected to osmotic stress for 4 h, and each leaf was used for transcriptomic and proteomic analyses. A Number correlations between proteins and mRNAs and between DAPs and DEGs. B Expression correlations between proteins and mRNA and between DAPs and DEGs. NDEGs, no differences in gene expression; NDAPs, no differences in protein expression. C Classification distribution map of term associated genes significantly enriched in GO and KEGG pathway. The numbers in parentheses represent the number of proteins associated with the term, and the color of the heat map represents the proportion of the protein associated with the GO and KEGG terms. Supplementary Fig. 4. DEGs and DAPs involved in photosynthesis, photorespiration and Chl metabolism in different-age maize leaves under osmotic stress. Ten-d-old seedlings were subjected to osmotic stress for 4 h, and each leaf was used for transcriptomic and proteomic analyses. A Light reaction. B Calvin cycle. C DEGs involved in Chl biosynthesis and degradation [file 44154_2024_159_MOESM1_ESM.pdf]

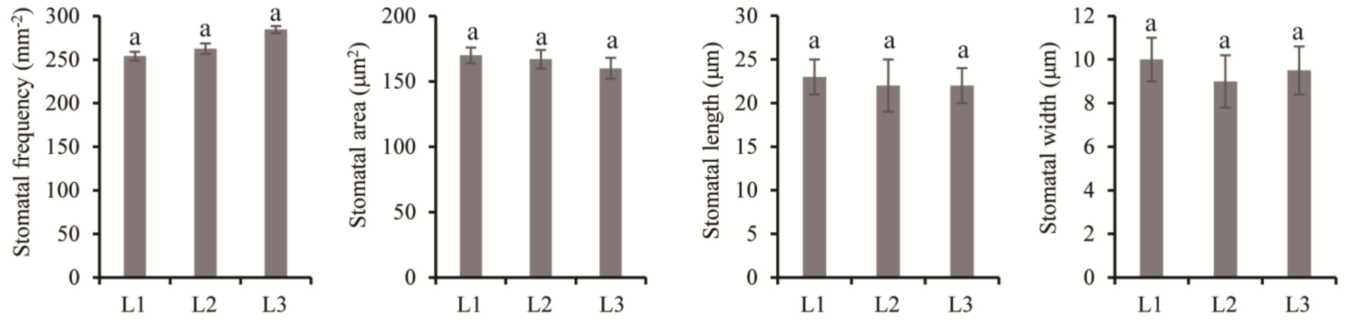

Supplementary Fig. 1. Comparison of stomata characteristics of different leaves. The abaxial surfaces of leaves in 10-d-old maize seedlings were observed under a microscope.

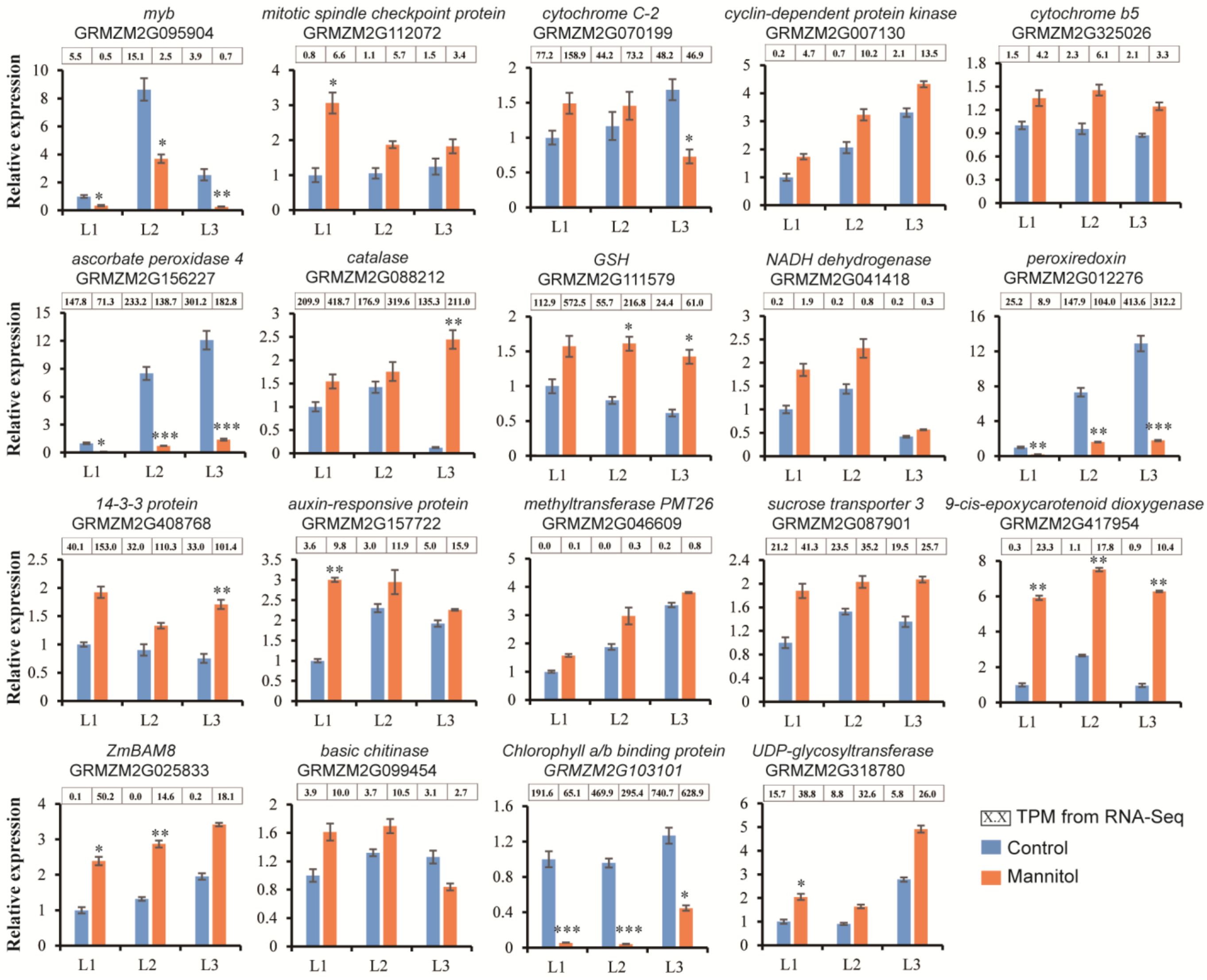

Supplementary Fig. 2 Reverse transcription quantitative-PCR (RT-qPCR) validation of expression changes of 19 DEGs in different maize leaves. Ten-d-old seedlings were subjected to osmotic stress for 4 h, and each leaf was used for RNA extraction and PCR analysis (triplicate). Mean relative expression levels of DEGs were normalized to a value of 1.0 in L1 under control condition with *ZmUBI* as a reference gene. Error bars indicated the SE values of three biological replicates. Asterisks show significant difference in expression changes as assessed by Student's t-test (* $P < 0.05$, ** $P < 0.01$, *** $P < 0.001$).

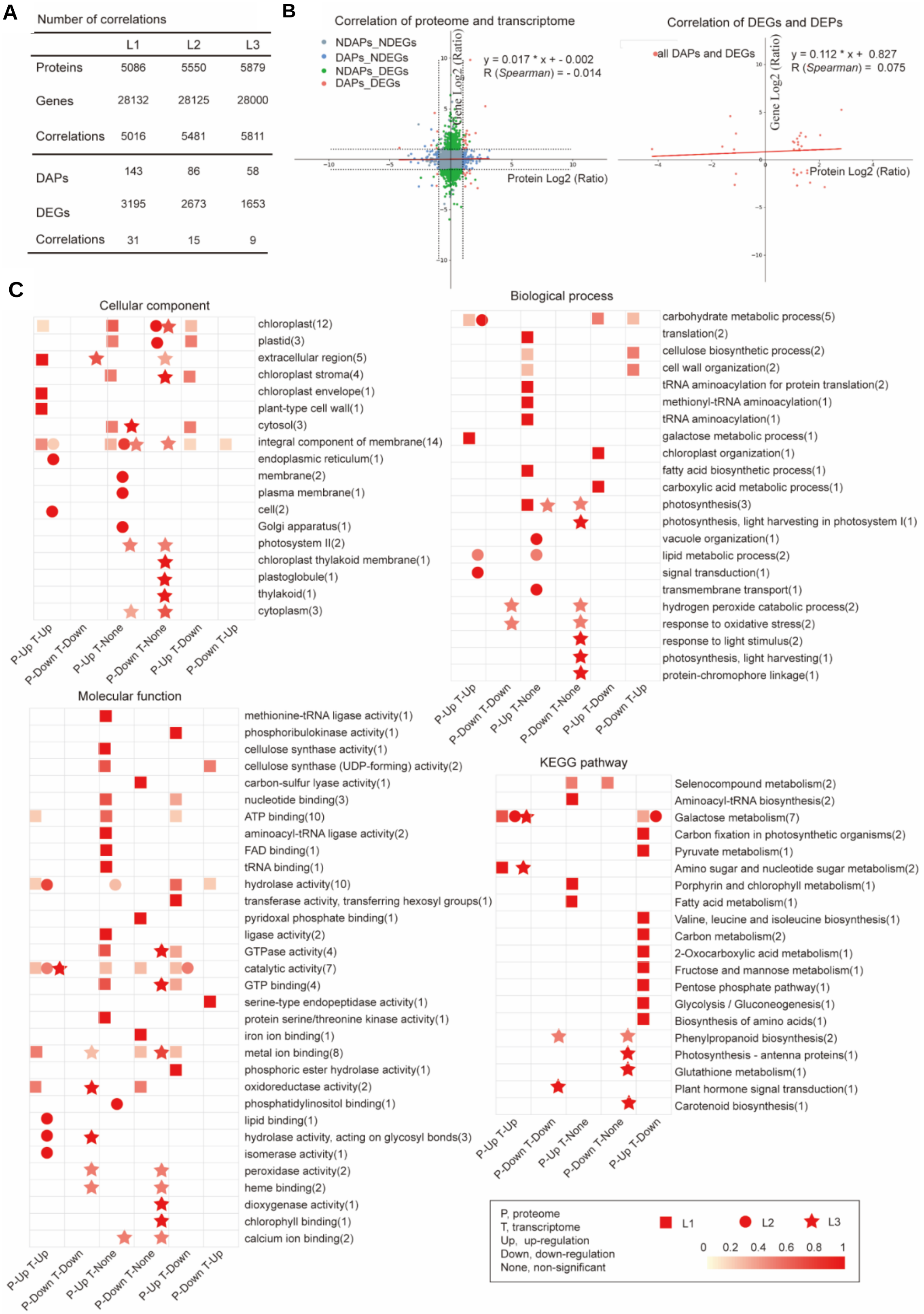

Supplementary Fig. 3 Correlation analyses of transcriptomic and proteomic data sets in different maize leaves under control and osmotic stress conditions. Ten-d-old seedlings were subjected to osmotic stress for 4 h, and each leaf was used for transcriptomic and proteomic analyses. **A** Number correlations between proteins and mRNAs and between DAPs and DEGs. **B** Expression correlations between proteins and mRNA and between DAPs and DEGs. NDEGs, no differences in gene expression; NDAPs, no differences in protein expression. **C** Classification distribution map of term associated genes significantly enriched in GO and KEGG pathway. The numbers in parentheses represent the number of proteins associated with the term, and the color of the heat map represents the proportion of the protein associated with the GO and KEGG terms.

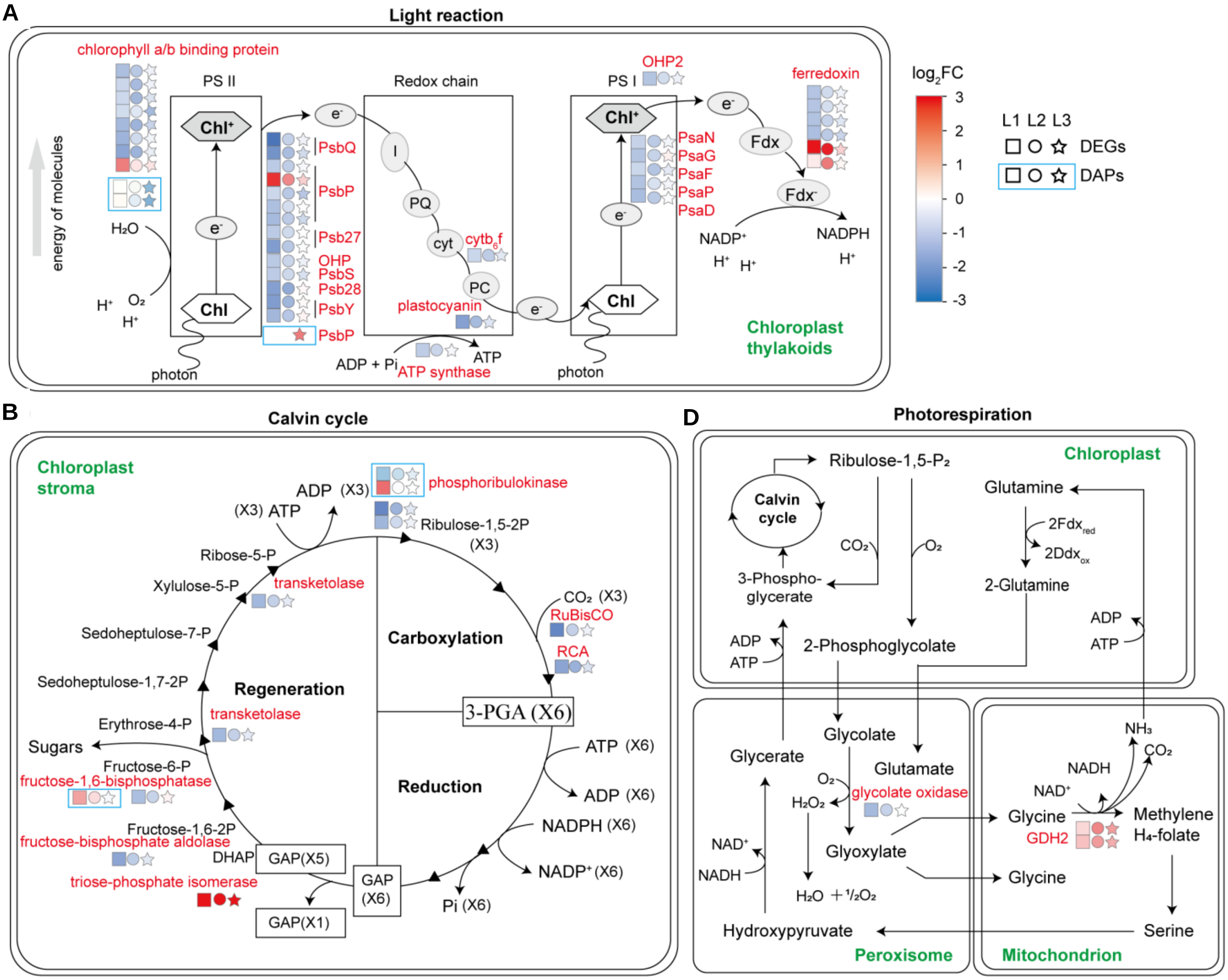

C

Chlorophyll biosynthesis

Gene symbol	L1	L2	L3	Name
GRMZM2G177412	-1.37	-0.82	-0.66	glutamyl-tRNA reductase
GRMZM2G127421	-1.06	-0.60	-0.31	chlorophyllase-2
GRMZM2G084958	-2.02	-1.20	-0.70	protochlorophyllide reductase 1
GRMZM2G063048	-1.28	-1.45	-0.83	Divinyl chlorophyllide a 8-vinyl-reductase
GRMZM2G036455	-1.05	-1.18	-0.75	protochlorophyllide reductase B
GRMZM2G323024	-1.10	-1.12	-1.06	Magnesium-chelatase subunit ChlH
GRMZM2G043453	-1.13	-0.66	-0.26	magnesium-chelatase subunit ChlD
GRMZM2G419806	-1.06	-0.93	-0.50	oil yellow 1

Chlorophyll degradation

Gene symbol	L1	L2	L3	Name
GRMZM2G339563	2.08	1.60	1.30	*pheophorbide a oxygenase*
GRMZM2G103881	4.60	2.99	2.45	*senescence-inducible chloroplast stay-green protein 1*
GRMZM2G091837	1.68	1.30	1.36	*senescence-inducible chloroplast stay-green protein 2*

Supplementary Fig. 4 DEGs and DAPs involved in photosynthesis, photorespiration and Chl metabolism in different-age maize leaves under osmotic stress. Ten-d-old seedlings were subjected to osmotic stress for 4 h, and each leaf was used for transcriptomic and proteomic analyses. **A** Light reaction. **B** Calvin cycle. **C** DEGs involved in Chl biosynthesis and degradation. **D** Photorespiration.

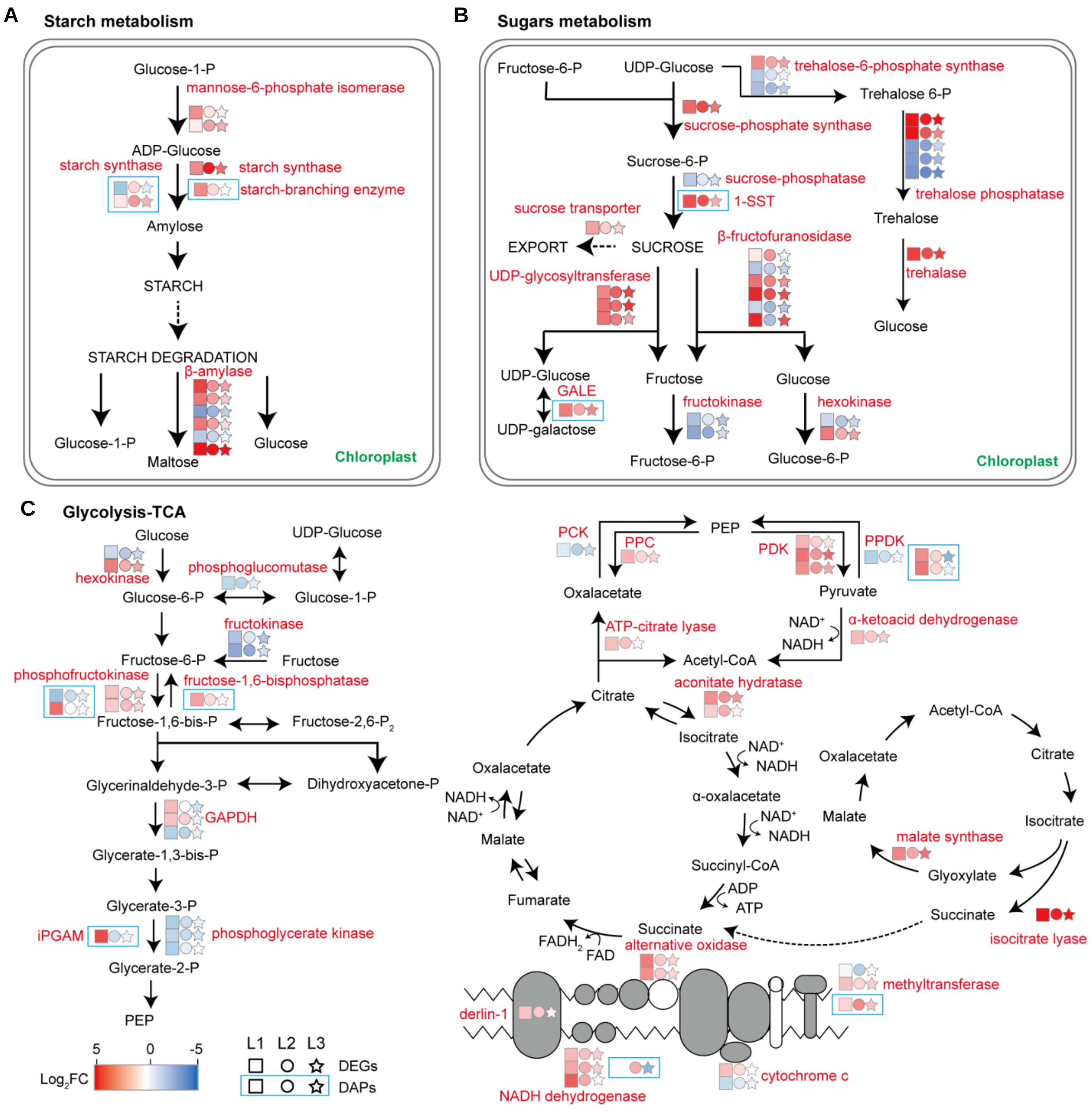

Supplementary Fig. 5 DEGs and DAPs involved in starch and sugar metabolism and glycolysis in different maize leaves under osmotic stress. Ten-d-old seedlings were subjected to osmotic stress for 4 h, and each leaf was used for analyses. **A** starch metabolism. **B** Sugars metabolism. **C** Glycolysis-TCA.

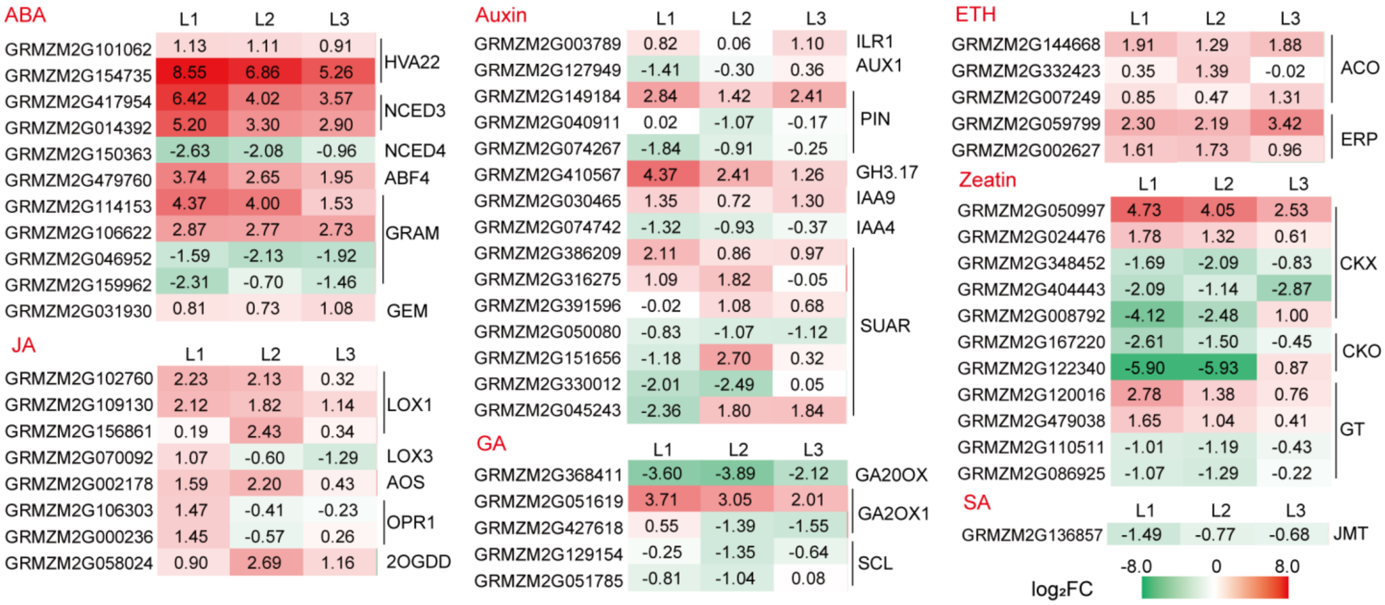

Supplementary Fig. 6 The transcript levels of DEGs related to phytohormones in different maize leaves under osmotic stress. Ten-d-old seedlings were subjected to osmotic stress for 4 h, and each leaf was used for analyses. Abbreviations: 2OGDD, 2-oxoglutarate-dependent dioxygenase; ACO, 1-aminocyclopropane-1-carboxylate oxidase; ABF4, ABRE binding factor 4; AUX1, auxin transporter 1; AOS, allene oxide synthase; CKO, CK oxidase; CKX, CK oxidase/dehydrogenase; ERP, ethylene-responsive protein; GA20OX1, GA20 oxidase; GA2OX1, GA2 oxidase 1; GEM, GL2 expression modulator; GH3.17, IAA-amido synthetases; GRAM, GRAM domain-containing protein; GT, glycosyltransferase; HVA22, abscisic acid-responsive HVA22 family protein; ILR1, IAA-leucine resistant 1; LOX, Lipoxygenase; NCED, nine-cis-epoxycarotenoid dioxygenase; JMT, jasmonic acid carboxyl methyltransferase; OPR1, 12-oxophytodienoate reductase 1; PIN, PIN-formed; SUAR, auxin-responsive SAUR family protein; SCL, scarecrow-like transcription factor. Data represent means ± SD of three biological replicates. Significant differences in expression levels are indicated with different letters ($P < 0.05$, ANOVA).

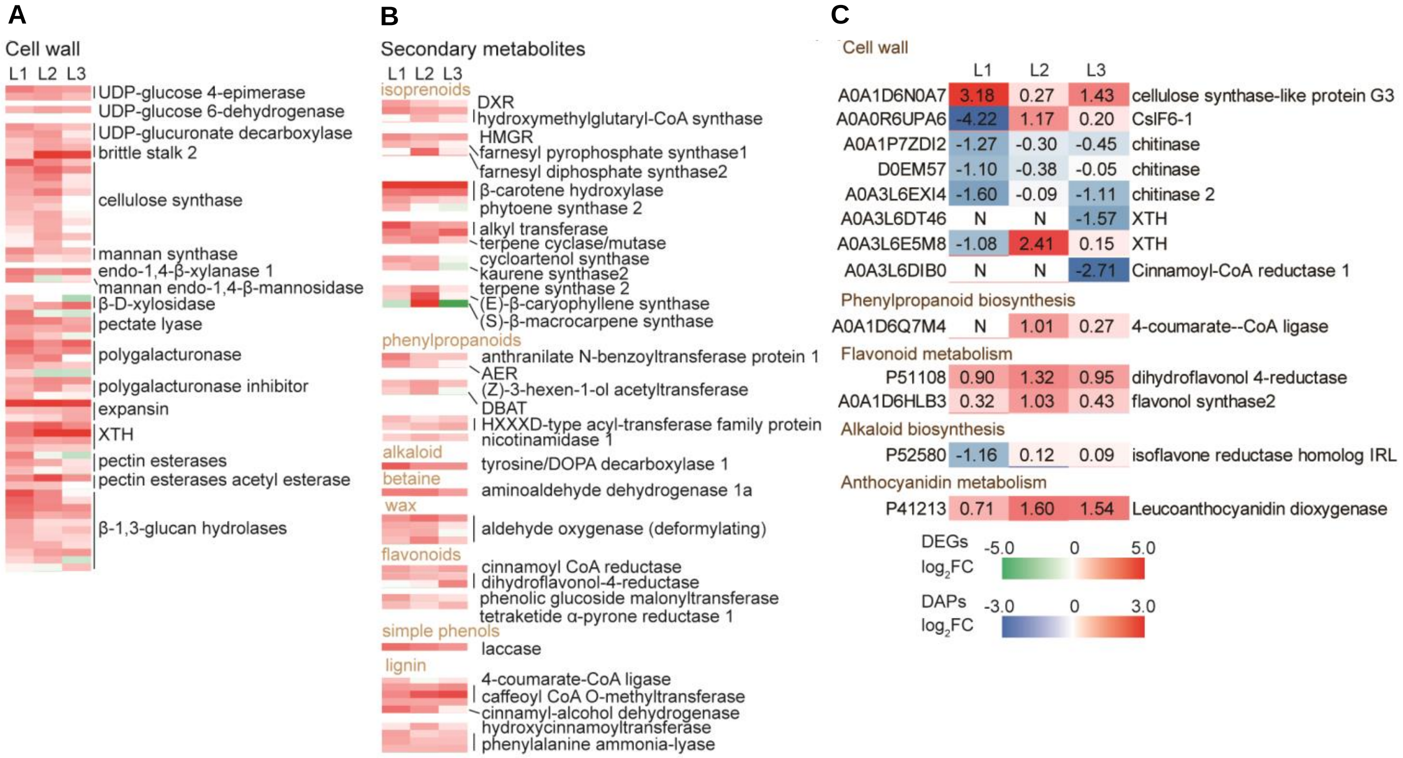

Supplementary Fig. 7 DEGs related to secondary metabolism in different maize leaves under osmotic stress. Ten-d-old seedlings were subjected to osmotic stress for 4 h, and each leaf was used for analyses. A DEGs related to wall synthesis and degradation. B DEGs related to various secondary metabolites. C DAPs related to secondary metabolism. Abbreviations: DBAT, 10-deacetylbaccatin III 10-O-acetyltransferase; DXR, 1-deoxy-D-xylulose-5-phosphate reductoisomerase; HMGR, 3-hydroxy-3-methylglutaryl-coenzyme A reductase; XTH, xyloglucan endotransglucosylase/hydrolase.

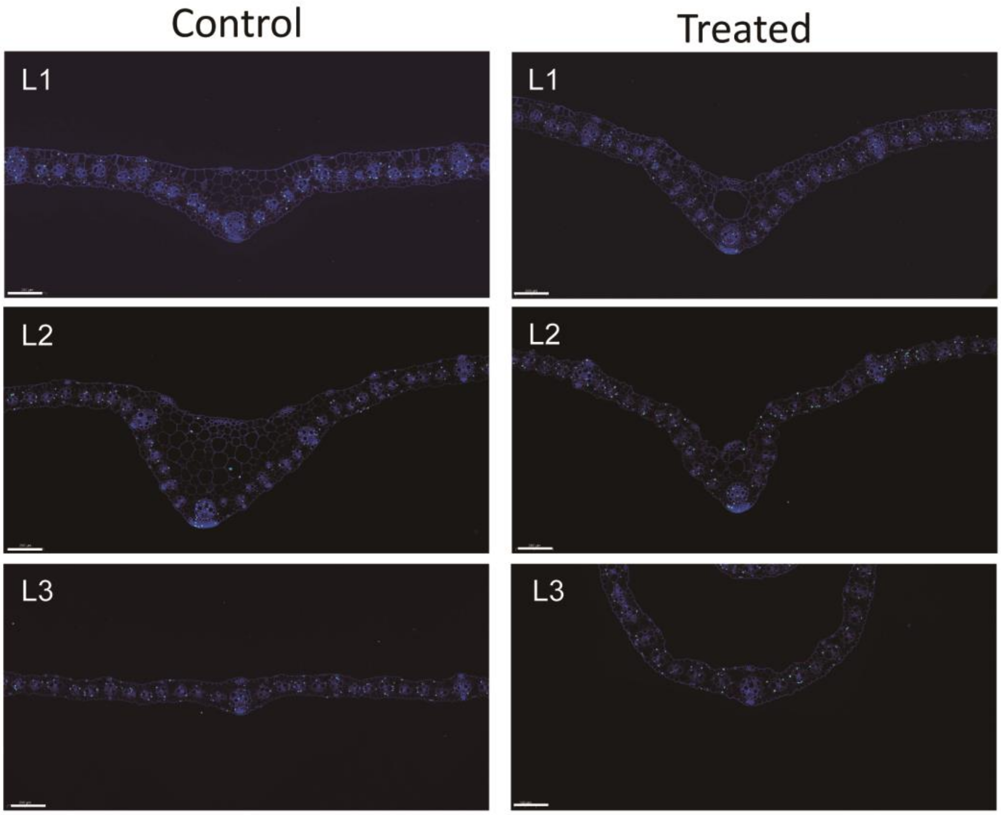

Supplementary Fig. 8 Programmed cell death (PCD) detection in different maize leaves under osmotic stress. Ten-d-old seedlings were subjected to osmotic stress for 4 h, and each leaf was used for analyses. PCD was detected using Fluorescein (FITC) Tunel Cell Apoptosis Detection Kit (Servicebio, Wuhan, China). The blue signal represents staining with propidium iodide (PI), green signal represents TUNEL-positive nuclei of dead cells due to PCD. The images were detected with a Zeiss LSM880 confocal laser scanning microscope. Scale bars, 100 μm.
