# Supplementary material for: Maize multi-omics reveal leaf water status controlling of differential transcriptomes, proteomes and hormones as mechanisms of age-dependent osmotic stress response in leaves
Source: Stress Biol. 2024 Mar 18;4(1):19. doi: 10.1007/s44154-024-00159-9 (PMC10948690; doi:10.1007/s44154-024-00159-9)
Supplement: Supplementary file 2 — Additional file 2: Supplementary Table 1. Primers used in this study. [file 44154_2024_159_MOESM2_ESM.docx]

**Supplementary Table 1** Primers used in this study.

| **Gene ID** | **Name** | **Left Primer** | **Right Primer** |
| --- | --- | --- | --- |
| GRMZM2G027378 | *ZmUBI* | TGCGTTAATCACGAGACAGG | AATCACAAAGACAGGCAGGG |
| GRMZM2G025833 | *ZmBAM8* | ACTACCTCGGCAACACCATC | GTGTCCTCAGGCCAGTTGTT |
| GRMZM2G414252 | *protoporphyrinogen oxidase* | TGGAGTTCGACCTGCTGAAT | CGTGCTGCTTCTTTGGGTAC |
| GRMZM2G095904 | *myb* | GTCATCCAACTCCACCAA | GTTCTTGATCTCGTTGTCC |
| GRMZM2G325026 | *cytochrome b5* | TATCATCATCTCACTTGTCATT | ATCCAACAGTCCTTCCTT |
| GRMZM2G156227 | *ascorbate peroxidase 4* | GACTGACTATGAGGTTGAC | TATGTGTAGGCTTCGTAGT |
| GRMZM2G111579 | *GSH1* | GCCTACTCTTACCGATTGG | TCAGCACCTCTCATCTCA |
| GRMZM2G088212 | *catalase* | GCAGAGAATGAACAGATT | TCAGCATAGGAGAAGATT |
| GRMZM2G012276 | *peroxiredoxin* | GAACTTCACCCTCAAGGA | GGTAGAAGTAAACCACGAC |
| GRMZM2G007130 | *cyclin-dependent protein kinase* | CTGGATCATCAAGACCAC | TTGAACCAGGAAGCGATC |
| GRMZM2G112072 | *mitotic spindle checkpoint protein* | AGATGACGCCAGAAGTAG | CATCAGCAAGCATCACAA |
| GRMZM2G070199 | *cytochrome C-2* | GCAGGCTACTCCTACTCT | GGTTAAGCAGGTACTCATACA |
| GRMZM2G041418 | *NADH dehydrogenase* | CGAGGCAAACGAAATCTT | TCCATACTTTGACAGGTGAT |
| GRMZM2G103101 | *Chlorophyll a/b binding protein* | AGCAGAGGAAGGGACATTCA | GGCATGAGTTACGGATGCTT |
| GRMZM2G087901 | *sucrose transporter 3* | CATGCGCTCACTTCATTCAT | CTTCGTGTCCCCTAGAGCAG |
| GRMZM2G318780 | *UDP-glycosyltransferase* | AGGTTGGGGTGATACAGCAG | GCACGGACTTGGTCCAGTAT |
| GRMZM2G157722 | *auxin-responsive protein* | ATTCAGGCGGTCAGTAATGG | GCTCTTGCCCATAGAATCCA |
| GRMZM2G417954 | *9-cis-epoxycarotenoid dioxygenase* | CAGATCGTGTTCAAGCTCCA | AGAGGTGGAAGCAGAAGCAG |
| GRMZM2G408768 | *14-3-3-like protein* | GTCTGGTGCAGAGAGGAAGG | AGGGGAGTTCAGGATCTCGT |
| GRMZM2G046609 | *dehydration-responsive protein* | GCACTGGAGGAAGGTAGTCG | CGCACCAGTCATGGTACATC |
| GRMZM2G099454 | *basic chitinase* | GCGCCTTCTACACCTACGAC | GTGGGTCCGTCTGTTTCTGT |
