# Supplementary material for: Maize multi-omics reveal leaf water status controlling of differential transcriptomes, proteomes and hormones as mechanisms of age-dependent osmotic stress response in leaves
Source: Stress Biol. 2024 Mar 18;4(1):19. doi: 10.1007/s44154-024-00159-9 (PMC10948690; doi:10.1007/s44154-024-00159-9)

**Supplementary Dataset 1** Gene sequence assembly, quality check, alternative splicing, and expression levels of maize Chang7-2t leaf transcriptome. RNA-seq analysis was performed on the first (L1), second (L2), and third (L3) leaves of maize drought-tolerant inbred line Chang7-2t under osmotic stress and control groups from three independent biological replicates. Raw reads of 143.74, 141.31, 141.99, 136.19, 142.02, and 144.78 Mb were generated from L1, L2 and L3 under control and osmotic stress, respectively. After removal of the adapters, low-quality sequences, and ambiguous reads, clean reads of 134.33, 133.27, 134.57, 130.06, 133.07, and 132.89 Mb were obtained (Dataset S1a). The Q30 scores of all libraries were above 89%, with an average of 90.19%, indicating reliable sequencing results (Dataset S1a). On average, 78.50% of the reads were mapped to the maize reference genome sequence (B73_RefGen_v4) using HISAT (Hierarchical Indexing for Spliced Alignment of Transcripts).

**Dataset S1a** Overview of the transcriptome sequencing and assembly.

| **Sample** | **Total Raw Reads (M)** | **Total Clean Reads (M)** | **Total Clean Bases (Gb)** | **Q30 (%)** | **Clean Reads Ratio (%)** | **Total Mapping (%)** | **Uniquely Mapping (%)** |
| --- | --- | --- | --- | --- | --- | --- | --- |
| C_L1_r1 | 47.33 | 44.63 | 6.7 | 90.41 | 94.31 | 78.5 | 76.01 |
| C_ L1_r2 | 47.33 | 44.72 | 6.71 | 90.38 | 94.48 | 79.23 | 76.6 |
| C_ L1_r3 | 49.08 | 44.98 | 6.75 | 90.47 | 91.65 | 79.64 | 77.08 |
| C_L2_r1 | 47.33 | 44.93 | 6.74 | 89.97 | 94.93 | 79.1 | 76.51 |
| C_ L2_r2 | 46.65 | 43.71 | 6.56 | 90.47 | 93.71 | 78.9 | 76.24 |
| C_ L2_r3 | 47.33 | 44.63 | 6.69 | 90.02 | 94.3 | 78.92 | 76.34 |
| C_L3_r1 | 47.33 | 44.67 | 6.7 | 89.75 | 94.38 | 78.92 | 76.22 |
| C_L3_r2 | 47.33 | 45.11 | 6.77 | 89.9 | 95.31 | 79.14 | 76.38 |
| C_L3_r3 | 47.33 | 44.79 | 6.72 | 90.28 | 94.65 | 79.47 | 76.76 |
| D_L1_r1 | 47.33 | 44.69 | 6.7 | 89.84 | 94.42 | 77.44 | 75.15 |
| D_ L1_r2 | 44.5 | 40.9 | 6.13 | 90.33 | 91.91 | 78.47 | 76.15 |
| D_ L1_r3 | 47.33 | 44.47 | 6.67 | 90.19 | 93.96 | 78.75 | 76.34 |
| D_L2_r1 | 47.33 | 44.97 | 6.75 | 90.24 | 95.02 | 79 | 76.37 |
| D_ L2_r2 | 45.61 | 43.42 | 6.51 | 89.91 | 95.21 | 78.49 | 76.1 |
| D_ L2_r3 | 49.08 | 44.68 | 6.7 | 90.67 | 91.03 | 78.59 | 75.95 |
| D_ L3_r1 | 49.08 | 44.97 | 6.75 | 90.18 | 91.63 | 78.97 | 76.32 |
| D_ L3_r2 | 49.08 | 44.89 | 6.73 | 90.29 | 91.47 | 79.25 | 76.68 |
| D_ L3_r3 | 46.62 | 43.03 | 6.45 | 90.13 | 92.3 | 79.2 | 76.46 |

Note: C, control group. D, 0.3 M mannitol osmotic stress group. r1, r2 and r3 indicate the three biological replicates. L1, L2 and L3 were designed from down to top in 10-d-old maize seedlings.

**Dataset S1b** Alternative splicing events of 18 RNA-seq samples compared to maize reference genome sequence. Using rMATS, five alternative splicing events were detected in the samples, including skipped exon (SE), alternative 5'-splicing site (A5SS), alternative 3'-splicing site (A3SS), mutually exclusive exons (MXE), and retained intron (RI).

| **Sample** | **A5SS** | **A3SS** | **MXE** | **RI** | **SE** | **Total** |
| --- | --- | --- | --- | --- | --- | --- |
| C_L1_r1 | 666 | 1637 | 106 | 756 | 3426 | 6591 |
| C_ L1_r2 | 744 | 1724 | 124 | 775 | 3988 | 7355 |
| C_ L1_r3 | 714 | 1703 | 118 | 787 | 3761 | 7083 |
| C_L2_r1 | 670 | 1575 | 98 | 735 | 3262 | 6340 |
| C_ L2_r2 | 720 | 1669 | 108 | 767 | 3293 | 6557 |
| C_ L2_r3 | 698 | 1625 | 123 | 741 | 3467 | 6654 |
| C_L3_r1 | 717 | 1680 | 110 | 767 | 3201 | 6475 |
| C_L3_r2 | 697 | 1644 | 119 | 746 | 3076 | 6282 |
| C_L3_r3 | 702 | 1621 | 100 | 735 | 3135 | 6293 |
| D_L1_r1 | 661 | 1534 | 115 | 679 | 4033 | 7022 |
| D_ L1_r2 | 682 | 1611 | 136 | 733 | 4137 | 7299 |
| D_ L1_r3 | 721 | 1666 | 169 | 768 | 4309 | 7633 |
| D_L2_r1 | 738 | 1731 | 135 | 793 | 3837 | 7234 |
| D_ L2_r2 | 735 | 1657 | 126 | 757 | 3800 | 7075 |
| D_ L2_r3 | 745 | 1682 | 147 | 789 | 4015 | 7378 |
| D_ L3_r1 | 748 | 1676 | 121 | 803 | 3573 | 6921 |
| D_ L3_r2 | 714 | 1667 | 103 | 754 | 3228 | 6466 |
| D_ L3_r3 | 647 | 1604 | 90 | 702 | 2997 | 6040 |

Note: C, control group. D, 0.3 M mannitol osmotic stress group. r1, r2 and r3 indicate the three biological replicates. L1, L2 and L3 were designed from down to top in 10-d-old maize seedlings. SE, skipped exon; A5SS, alternative 5 'splicing site; A3SS, alternative 3' splicing site; MXE, mutually exclusive exons; RI, retained intron.

**Dataset S1c** Transcript expression levels of 18 samples from leaves of three ages. *Upper*, box diagram. *Down*, stacking diagrams. C, control group. D, 0.3 M mannitol osmotic stress group. r1, r2 and r3 indicate the three biological replicates. 1st_leaf, L1; 2nd_leaf, L2; 3rd_leaf, L3, were designed from down to top in 10-d-old maize seedlings. TPM (transcripts per million) values were used to evaluate the gene expression levels in this study. The box and stacking diagram of the expression level suggested that the median and quartile values among the samples being compared for differential expression were almost identical. The percentage of genes with TPM < 1 in the 18 samples ranged from 32.72% to 33.93%, and the percentage of genes with TPM ≥ 10 in the 18 samples ranged from 36.54% to 41.03%.


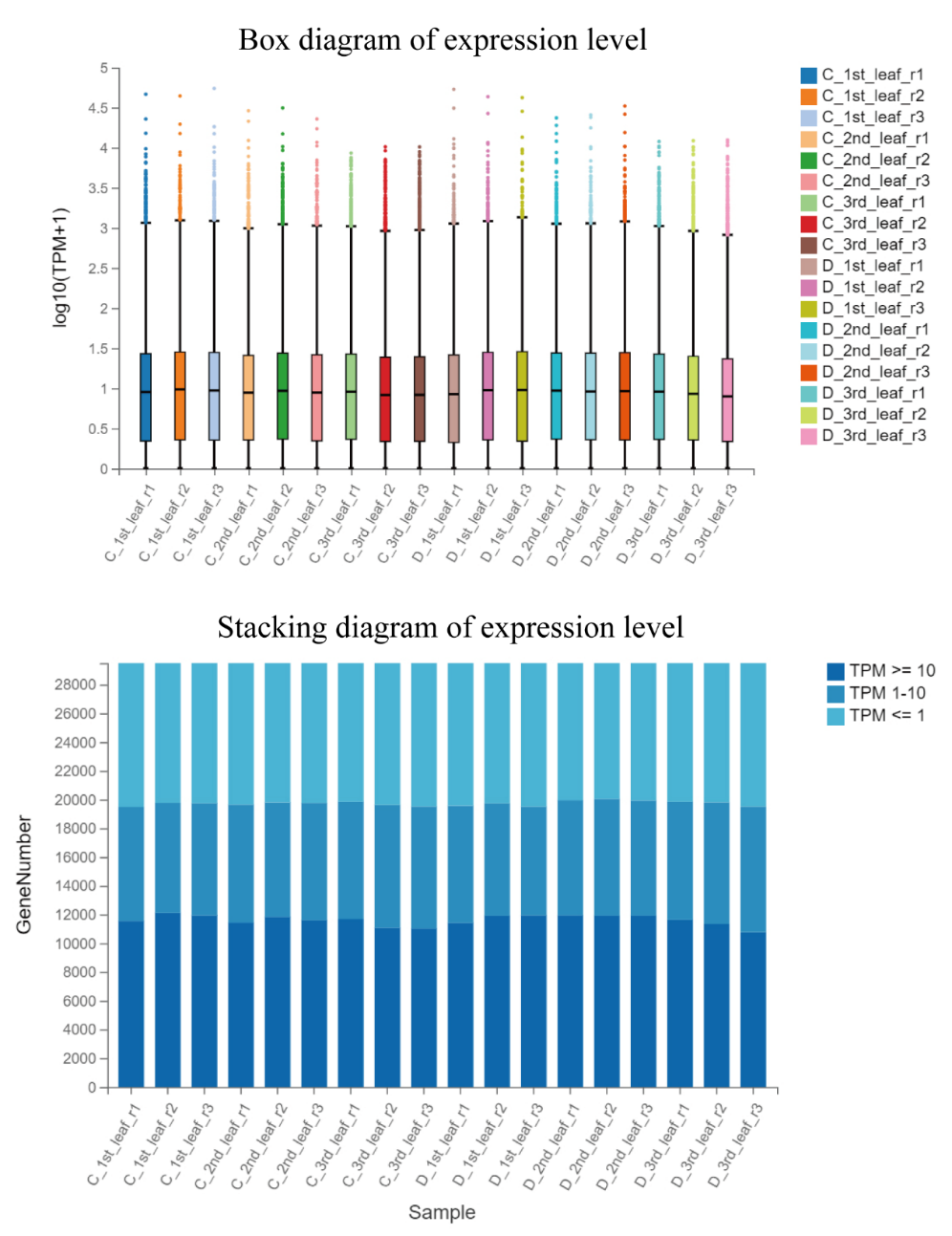

Supplement: Supplementary file 3 — Additional file 3: Supplementary Dataset 1. Gene sequence assembly, quality check, alternative splicing, and expression levels of maize leaf transcriptomes. [file 44154_2024_159_MOESM3_ESM.docx]
